# Laboratory Evolution of a *Saccharomyces cerevisiae* × *S. eubayanus* Hybrid Under Simulated Lager-Brewing Conditions

**DOI:** 10.3389/fgene.2019.00242

**Published:** 2019-03-29

**Authors:** Arthur R. Gorter de Vries, Maaike A. Voskamp, Aafke C. A. van Aalst, Line H. Kristensen, Liset Jansen, Marcel van den Broek, Alex N. Salazar, Nick Brouwers, Thomas Abeel, Jack T. Pronk, Jean-Marc G. Daran

**Affiliations:** ^1^Industrial Microbiology, Department of Biotechnology Delft, Delft University of Technology, Delft, Netherlands; ^2^Delft Bioinformatics Lab, Department of Intelligent Systems, Delft University of Technology, Delft, Netherlands; ^3^Infectious Disease and Microbiome Program, Broad Institute of MIT and Harvard, Boston, MA, United States

**Keywords:** *Saccharomyces pastorianus*, loss of heterozygosity, laboratory evolution, domestication, maltotriose utilization, flocculation

## Abstract

*Saccharomyces pastorianus* lager-brewing yeasts are domesticated hybrids of *S. cerevisiae* x *S. eubayanus* that display extensive inter-strain chromosome copy number variation and chromosomal recombinations. It is unclear to what extent such genome rearrangements are intrinsic to the domestication of hybrid brewing yeasts and whether they contribute to their industrial performance. Here, an allodiploid laboratory hybrid of *S. cerevisiae* and *S. eubayanus* was evolved for up to 418 generations on wort under simulated lager-brewing conditions in six independent sequential batch bioreactors. Characterization of 55 single-cell isolates from the evolved cultures showed large phenotypic diversity and whole-genome sequencing revealed a large array of mutations. Frequent loss of heterozygosity involved diverse, strain-specific chromosomal translocations, which differed from those observed in domesticated, aneuploid *S. pastorianus* brewing strains. In contrast to the extensive aneuploidy of domesticated *S. pastorianus* strains, the evolved isolates only showed limited (segmental) aneuploidy. Specific mutations could be linked to calcium-dependent flocculation, loss of maltotriose utilization and loss of mitochondrial activity, three industrially relevant traits that also occur in domesticated *S. pastorianus* strains. This study indicates that fast acquisition of extensive aneuploidy is not required for genetic adaptation of *S. cerevisiae* × *S. eubayanus* hybrids to brewing environments. In addition, this work demonstrates that, consistent with the diversity of brewing strains for maltotriose utilization, domestication under brewing conditions can result in loss of this industrially relevant trait. These observations have important implications for the design of strategies to improve industrial performance of novel laboratory-made hybrids.

## Introduction

*Saccharomyces* yeasts are popular eukaryotic models for studying genome hybridization, chromosome (mis)segregation and aneuploidy (Botstein et al., 1997; Sheltzer et al., 2011). The genus *Saccharomyces* arose between 10 and 20 million years ago and currently comprises eight described species, as well as interspecies hybrids (Liti et al., 2006; Hittinger, 2013; Naseeb et al., 2017). Absence of a prezygotic barrier between *Saccharomyces* species facilitates hybridization, although spore viabilities of the resulting hybrids is typically well below 10% (Liti et al., 2006; Hittinger, 2013; Naseeb et al., 2017). Several interspecies *Saccharomyces* hybrids are tightly associated with domestication in industrial processes. *S. pastorianus* lager-brewing yeasts are domesticated *S. cerevisiae* × *S. eubayanus* hybrids (Libkind et al., 2011). Double and triple hybrids between *S. cerevisiae, S. kudriavzevii* and *S. uvarum* are closely associated with wine fermentation (González et al., 2006; Querol and Bond, 2009; Marsit and Dequin, 2015). *S. bayanus* cider fermentation yeasts are domesticated *S. uvarum* × *S. eubayanus* hybrids (Naumov et al., 2001). Reconstruction of the corresponding *Saccharomyces* hybrids in the laboratory showed improved performance, relative to the parental species. For example, laboratory-made *S. cerevisiae* × *S. eubayanus* hybrids combined sugar utilization characteristics of *S. cerevisiae* and the superior performance at low temperatures of *S. eubayanus* (Hebly et al., 2015; Krogerus et al., 2017). Similarly, hybrids of *S. cerevisiae, S. kudriavzevii* and *S. uvarum* combined traits of their parental species relevant to industrial wine fermentation, such as flocculence, sugar utilization kinetics, stress tolerance and aroma production (Coloretti et al., 2006; Lopandic et al., 2016).

The relevance of laboratory hybridization of *Saccharomyces* species extends beyond reconstruction of existing, domesticated hybrids. The ability of hybridization to generate extensive phenotypic diversity has raised interest in the development of novel *Saccharomyces* hybrids for specific industrial processes (Krogerus et al., 2017). For example, an *S. cerevisiae* × *S. paradoxus* hybrid produced high concentrations of aromatic compounds that are of interest for wine making (Bellon et al., 2011). Hybrids between *S. cerevisiae* and *S. arboricola* or *S. mikatae* were able to utilize the sugars in wort at low temperatures and produced particularly aromatic beer (Nikulin et al., 2017). Laboratory hybrids of *S. cerevisiae* and *S. kudriavzevii* or *S. mikatae* yielded xylose-consuming strains with high inhibitor tolerance for second generation biofuel production (Peris et al., 2017).

The alloeuploid genomes of laboratory hybrids of *Saccharomyces* species strongly differ from the extremely aneuploidy genomes of the domesticated strains used in traditional industrial processes. For example, the genomes of *S. pastorianus* lager-brewing yeasts contain between 45 and 79 chromosomes (Van Den Broek et al., 2015; Okuno et al., 2016), a degree of aneuploidy that is not observed elsewhere in the *Saccharomyces* genus (Gorter de Vries et al., 2017b). However, it remains unclear when and how domestication resulted in the extensive chromosome copy number variations and phenotypic diversity of current *S. pastorianus* strains.

Hybrid genomes have a well-documented increased tendency to become aneuploid due to an increased rate of chromosome missegregation during mitosis and/or meiosis (Chambers et al., 1996; Liti et al., 2006). Aneuploidy reduces the efficiency of sporulation and can thereby complicate genetic modification, impeding breeding and targeted strain improvement (Santaguida and Amon, 2015; Gorter de Vries et al., 2017a). In evolutionary contexts, aneuploidy is generally seen as a transient adaptation mechanism, whose positive impacts are eventually taken over by more parsimonious mutations (Yona et al., 2012). When grown mitotically, sporulated hybrid strains were prone to further chromosome missegregation resulting in more extensive chromosome copy number variations (Lopandic et al., 2016). Even genomes of *Saccharomyces* hybrids that had not undergone meiosis displayed increased rates of mitotic chromosome missegregation during mitosis (Delneri et al., 2003). Indeed, when evolved in lignocellulosic hydrolysates, cultures of *S. cerevisiae* × *S. kudriavzevii* and *S. cerevisiae* × *S. mikatae* hybrids exhibited segmental and full-chromosome aneuploidy after only 50 generations (Peris et al., 2017). Similarly, when evolved under wine fermentation conditions, *S. cerevisiae* × *S. kudriavzevii* hybrids displayed extensive genome reorganizations that led to a significant reduction of their genome content (Pérez Través et al., 2014).

Genetic instability of hybrid genomes could be detrimental to stable, robust industrial performance. Therefore, to assess industrial applicability of new hybrids generated in the laboratory, it is important to determine their genome stability under industrially relevant conditions. Moreover, laboratory evolution under simulated industrial conditions can increase understanding of the selective pressures that shaped the genomes of domesticated microorganisms (Bachmann et al., 2012; Gibbons et al., 2012; Gibbons and Rinker, 2015).

The goal of the present study was to investigate how the previously constructed allodiploid *S. cerevisiae* × *S. eubayanus* hybrid IMS0408 (Hebly et al., 2015) evolves under simulated lager-brewing conditions, with a specific focus on genome dynamics and on acquisition or loss of brewing-related phenotypes. To mimic successive lager beer fermentation processes, the hybrid strain was subjected to sequential batch cultivation on industrial wort, in six independent bioreactor setups. After up to 418 generations, the genotypic and phenotype diversity generated in these laboratory evolution experiments was analyzed by characterization of 55 single-cell isolates. After whole-genome resequencing of each isolate using 150 bp paired-end reads, sequence data were mapped to high-quality reference genomes of the parental strains to identify genomic changes. Phenotypic analysis of the isolates focused on the ability to utilize maltotriose, flocculation and the respiratory capacity. We interpreted these results in the context of the domestication history of *S. pastorianus* brewing strains as well as in relation to genome stability and industrial application of newly generated *Saccharomyces* hybrids.

## Materials and Methods

### Yeast Strains and Media

*Saccharomyces* strains used in this study are listed in Supplementary Table 1. Yeast strains and *E. coli* strains containing plasmids were stocked in 1 mL aliquots after addition of 30% v/v glycerol to the cultures and stored at −80°C. For preparation of stock cultures and inocula of bioreactors, yeast strains were routinely propagated in shake flasks containing 100 mL YPD (10 g.L^−1^ yeast extract, 20 g.L^−1^ yeast peptone and 20 g.L^−1^ glucose) at 30°C and 200 RPM in an Brunswick Innova43/43R shaker (Eppendorf Nederland B.V., Nijmegen, The Netherlands). For cultivation on solid media, YPD medium was supplemented with 20 g.L^−1^ Bacto agar (Becton Dickinson, Breda, The Netherlands) and incubation was done at 30 °C. Synthetic medium (SM), containing 3 g.L^−1^ KH_2_PO_4_, 0.5 g.L^−1^ MgSO_4_.7H_2_O, 5 g.L^−1^ (NH_4_)_2_SO_4_, 1 mL.L^−1^ of a trace element solution and 1 mL.L^−1^ of a vitamin solution, was prepared as previously described (Verduyn et al., 1992). SM maltotriose was supplemented with 20 g.L^−1^ of maltotriose and SM ethanol with 20 mL.L^−1^ of ethanol. Selection for the amdS marker was performed on SM-AC: SM with 0.6 g·L^−1^ acetamide and 6.6 g·L^−1^ K_2_SO_4_ instead of (NH_4_)_2_SO_4_ as nitrogen source (Solis-Escalante et al., 2013). For counter selection of the amdS marker, strains were first grown on YPD and then on SM-FAC: SM supplemented with 2.3 g·L^−1^ fluoroacetamide (Solis-Escalante et al., 2013). Industrial wort was provided by HEINEKEN Supply Chain B.V., Zoeterwoude, the Netherlands, and contained 14.4 g·L^−1^ glucose, 2.3 g·L^−1^ fructose, 85.9 g·L^−1^ maltose, 26.8 g·L^−1^ maltotriose and 269 mg/L free amino nitrogen. The wort was supplemented with 1.5 mg/L Zn^2+^ by addition of ZnSO_4_.7H_2_O, then autoclaved for 30 min at 121°C and, prior to use, filtered through Nalgene 0.2 μm SFCA bottle-top filters (ThermoFisher Scientific, Waltham, MA). For experiments performed with diluted wort, two volumes of sterile demineralized water were added per volume of wort. To prevent excessive foaming during the aeration phase of the bioreactor experiments, (un)diluted wort was supplemented with 0.2 mL.L^−1^ of sterile Pluronic PE 6100 antifoam (Sigma-Aldrich, Zwijndrecht, the Netherlands).

### Analytical Methods and Statistics

Optical density at 660 nm was measured with a Libra S11 spectophotometer (Biochrom, Cambridge, UK). HPLC analysis of sugar and metabolite concentrations was performed with an Agilent Infinity 1260 chromatography system (Agilent Technologies, Santa Clara, CA) with an Aminex HPX-87 column (Bio-Rad, Lunteren, The Netherlands) at 65°C, eluted with 5 mM H_2_SO_4_. Significance of data was assessed by an unpaired two-tailed Student's t-test with a 95% confidence interval.

### Laboratory Evolution and Single Colony Isolation

The hybrid yeast strain IMS0408 was evolved under three different conditions in duplicate in Minifors 2 bioreactors (INFORS HT, Velp, the Netherlands) with a working volume of 100 mL: on diluted wort at 30°C (LG30.1 and LG30.2), on diluted wort at 12°C (LG12.1 and LG12.2) and on full-strength wort at 12°C (HG12.1 and HG12.2). Sequential batch cultivation was performed with 10 and 30 mL.min^−1^ of headspace N_2_ flushing at 12 and 30°C, respectively. The percentage of CO_2_ in the outlet gas stream, the culture pH and the dissolved oxygen concentration in the broth were continuously monitored. The end of a batch cultivation cycle was automatically triggered when the percentage of CO_2_ in the offgas decreased below 75 and 10% of the maximum value reached during that cycle for growth on diluted wort and full-strength wort, respectively. These CO_2_ percentages correspond to the moment at which sugar utilization was complete in the first batch cycle for each condition, as determined by HPLC measurements. When the CO_2_ threshold was reached, the reactor was emptied while stirring at 1200 RPM leaving about 7 mL to inoculate the next batch. Upon addition of fresh medium, the broth was stirred at 500 RPM and sparged with 500 mL.min^−1^ of pressurized air during 5 min for diluted wort or 12 h for wort. During the remainder of each batch cultivation cycle, the medium was not sparged or stirred and the pH was not adjusted. LG30.1 and LG30.2 were carried out for 116 and 117 cycles, respectively, LG12.1 and LG12.2 were carried out for 29 cycles and HG12.1 and HG12.2 were carried out for 13 and 16 cycles, respectively. Culture samples from all six reactors were then streaked on YPD plates and after three subsequent restreaks, frozen stock cultures of single colony isolates were prepared. By default, five isolates were obtained for each culture. For LG12.1 and LG30.1, two different colony morphologies were observed, therefore five elevated and conically-shaped colonies and five regular flat colonies were stocked. The experiments at 12°C on diluted wort were continued for 4 months until a total of 58 and 57 cycles was reached for LG12.1 and LG12.2, respectively, and five single-cell isolates were obtained for each reactor as described above.

### Genomic DNA Extraction and Whole Genome Sequencing

Yeast cultures were incubated in 500-mL shake-flasks containing 100 mL YPD at 30°C on an orbital shaker set at 200 RPM until the strains reached stationary phase at an OD_660_ between 12 and 20. Genomic DNA was isolated using the Qiagen 100/G kit (Qiagen, Hilden, Germany) according to the manufacturer's instructions and quantified using a Qubit® Fluorometer 2.0 (ThermoFisher Scientific). For IMS0408 and the evolved isolates, genomic DNA was sequenced at Novogene Bioinformatics Technology Co., Ltd (Yuen Long, Hong Kong) on a HiSeq2500 sequencer (Illumina, San Diego, CA) with 150 bp paired-end reads using PCR-free library preparation.

### Genome Analysis

A high quality reference genome was constructed by combining near-complete assemblies of *S. cerevisiae* CEN.PK113-7D (Salazar et al., 2017) and *S. eubayanus* CBS 12357^T^ (Brickwedde et al., 2018). The kanMX marker present in IMS0408 was inserted as an additional contig (Wach et al., 1994). For each evolved strain, raw Illumina reads were aligned against the reference genome using the Burrows–Wheeler Alignment tool (BWA, version 0.7.15-r1142) and further processed using SAMtools (version 1.3.1) and Pilon (version 1.18) for variant calling (Li et al., 2009; Li and Durbin, 2010; Walker et al., 2014). SNPs and INDELs that were also called or which were ambiguous in IMS0408, were disregarded. Copy number was determined based on read coverage analysis. Chromosomal translocations were detected using Breakdancer (version 1.3.6) (Chen et al., 2009). Only translocations which were supported by at least 10% of the reads aligned at that locus and which were absent in strain IMS0408 were considered. All SNPs, INDELs, recombinations and copy number changes were manually confirmed by visualizing the generated.bam files in the Integrative Genomics Viewer (IGV) software (Robinson et al., 2011). A complete list of identified mutations is provided in Supplementary Data File 1. For chimeric open-reading-frame reconstruction, reads aligning within 3 kbp of an identified recombination site and their paired reads were extracted using Python and were assembled using SPAdes (Bankevich et al., 2012). The resulting contigs were aligned against ORFs of the genes affected by the recombination to identify the recombination point, and the complete recombined ORF was reconstructed. Original and recombined ORFs were then aligned and translated using CloneManager (version 9.51, Sci-Ed Software, Denver, CO) to determine whether the translocation had introduced frameshifts or premature stop codons.

### DNA Content Determination by Flow Cytometric Analysis

Exponential-phase shake flask cultures on YPD were diluted to an OD_660_ of 1. A 1 mL sample (~10^7^ cells) was then washed in cold demineralized water and resuspended in 800 μL 70% ethanol while vortexing. After addition of another 800 μL 70% ethanol, fixed cells were stored at 4°C until further staining and analysis. DNA was then stained with SYTOX Green as described previously (Haase and Reed, 2002). Samples were analyzed on a Accuri C6 flow cytometer (BD Biosciences, Franklin Lakes, NJ) equipped with a 488-nm laser and the median fluorescence of cells in the 1n and 2n phases of the cell cycle was determined using FlowJo (BD Biosciences). The 1n and 2n medians of strains CEN.PK113-7D (n), CEN.PK122 (2n), and FRY153 (3n) were used to create a standard curve of fluorescence versus genome size with a linear curve fit, as performed previously (Van Den Broek et al., 2015). The genome size of each tested strain was estimated by averaging predicted genome sizes of the 1n and 2n population in assays on three independent cultures.

### Identification of Strains With Respiratory Deficiency

Respiratory competence was assessed through their ability to grow on ethanol. Samples from 24 h shake-flask cultures on YPD (30°C, 200 RPM) were washed twice with demineralized water and used to inoculate duplicate aerobic shake flasks containing 100 mL of SM with 2% ethanol to an OD_660_ of 0.2. After 72 h incubation at 30°C and 200 RPM, OD_660_ was measured.

### Assay for Calcium-Dependence of Flocculation

Two 100 μL aliquots from overnight cultures on YPD were washed with sterile demineralized water. One aliquot was resuspended in demineralized water and the other in 50 mM EDTA (pH 7.0). Both samples were imaged at 100 x magnification under a Z1 microscope (Carl Zeiss BV, Breda, the Netherlands) to assess flocculence and its reversal by EDTA chelation of calcium ions.

### Plasmid Construction

All plasmids were propagated in *E. coli* DH5α (Table 1). The gRNAs to target *ScSFL1* and *SeSFL1* (Supplementary Table 2) were designed as previously described (Gorter de Vries et al., 2017a) and ordered as *de novo* synthesized plasmids pUD711 (*ScSFL1*) and pUD712 (*SeSFL1*) at GeneArt (ThermoFisher Scientific). Plasmid pUDP104, expressing gRNA_*ScSFL*1_ and *cas9*, was constructed by Golden Gate cloning by digesting pUDP004 and pUD711 using BsaI and ligating with T4 ligase (Engler et al., 2008). Similarly, plasmid pUDP105, expressing gRNA_*SeSFL*1_ and *cas9*, was constructed from pUDP004 and pUD712. Correct assembly was verified by restriction analysis using PdmI.

**Table 1:** Plasmids used throughout this study.

**Name**	**Relevant genotype**	**Origin**
pUD711	ori *bla* gRNA-*ScSFL1*	GeneArt™
pUD712	ori *bla* gRNA-*SeSFL1*	GeneArt™
pUDE481	ori *bla* ARS4/CEN6 hyg^R^ *ScTDH3*p-mTurquoise2-*ScADH1*t	(Gorter de Vries et al., 2018a)
pUDE482	ori *bla* ARS4/CEN6 hyg^R^ *ScTEF1*p-Venus-*ScENO2*t	(Gorter de Vries et al., 2018a)
pUDP004	ori *bla* panARSopt amdSYM *Spcas9*	(Gorter de Vries et al., 2017a)
pUDP045	ori *bla* panARSopt amdSYM *Spcas9* gRNA-*ScMAL11*	(Gorter de Vries et al., 2018a)
pUDP104	ori *bla* panARSopt amdSYM *Spcas9* gRNA-*ScSFL1*	This study
pUDP105	ori *bla* panARSopt amdSYM *Spcas9* gRNA-*SeSFL1*	This study

### Strain Construction

The S*cTEF1*p-Venus-*ScENO2*t repair fragment with flanks for homologous recombination in the *ScMAL11* locus was PCR amplified from plasmid pUDE482 using primers 12989 and 12990 (Supplementary Table 2). The *ScTDH3*p-mTurquoise2-*ScADH1*t repair fragment with flanks for homologous recombination in the *ScSFL1* locus was PCR amplified from plasmid pUDE482 using primers 13564 and 13565. The S*cTEF1*p-Venus-*ScENO2*t repair fragment with flanks for homologous recombination in the *SeSFL1* locus was PCR amplified from plasmid pUDE481 using primers 13566 and 13567.

All strains were transformed by electroporation as described previously, with 300 ng of gRNA/Cas9 expression plasmid and 1 μg of repair fragment (Gorter de Vries et al., 2017a). Strains IMX1698 (mVenus::Δ*Scmal11*), IMX1824 (mTurquoise2::Δ*Scsfl1*) and IMX1825 (Venus::Δ*Sesfl1*) were constructed by transforming IMS0408 with the appropriate repair fragments and plasmids pUDP045, pUDP104 and pUDP105, respectively. Strain IMX1826 (mTurquoise2::Δ*Scsfl1* Venus::Δ*Sesfl1*) was constructed by transforming IMX1824 using the appropriate repair fragment and plasmid pUDP105. After electroporation, cells were transferred to 20 mL SM-Ac medium to select successful transformants and incubated at 30°C for 3 to 5 days. After growth was observed, 200 μL of culture was transferred to 20 mL fresh SM-Ac and incubated similarly during 24h. Finally, 200 μL from the second culture was transferred to 20 mL fresh YPD medium to maximize expression of fluorescent proteins. Successfully gene-edited cells were sorted using the BD FACSAria™ II SORP Cell Sorter (BD Biosciences) as described previously (Gorter de Vries et al., 2018a). The plasmids were cured from strains IMX1698, IMX1824, IMX1825, and IMX1826 by subsequent growth on YPD and plating on SM-FAC. After confirmation of the correct genotype by colony PCR, randomly picked colonies were used to prepare frozen stocks.

### Biomass Sedimentation Assay

IMS0408, IMS0556, IMS0558, IMS0559, IMS0617, IMX1824, IMX1825, and IMX1826 were grown in triplicate during 72 h in vented 50 mL Bio-One Cellstar Cellreactor tubes (Sigma-Aldrich) on 20 mL YPD at 30°C and 200 RPM until stationary phase. For each sample, the biomass was resuspended by vigorous vortexing and 1 mL was sampled immediately after vortexing from right underneath the meniscus. After 60 s of stationary incubation, another sample was taken by the same procedure. Biomass sedimentation was quantified as the ratio of the OD_660_ values of the two samples.

### Evaluation of Maltotriose Fermentation

Each strain was grown microaerobically in 100 mL serum bottles containing 100 mL medium and shaken at 200 RPM. Medium (full-strength or diluted wort) and incubation temperature (12 or 30°C) were the same as in the evolution experiment from which a strain had been isolated. Strain IMS0408 was included as a control for each condition. Bottles were inoculated to an OD_660_ of 0.2 from aerobic shake-flask precultures grown on same medium and under the same conditions. During cultivation, 8 to 12 samples were taken at regular intervals for OD_660_ measurements and metabolite analysis by HPLC. When no further sugar consumption was recorded over an interval of at least 48h, the fermentation was considered finished.

## Results

### Simulating Domestication Under Lager-Brewing Conditions in Sequential Batch Bioreactors

**Figure 1:**
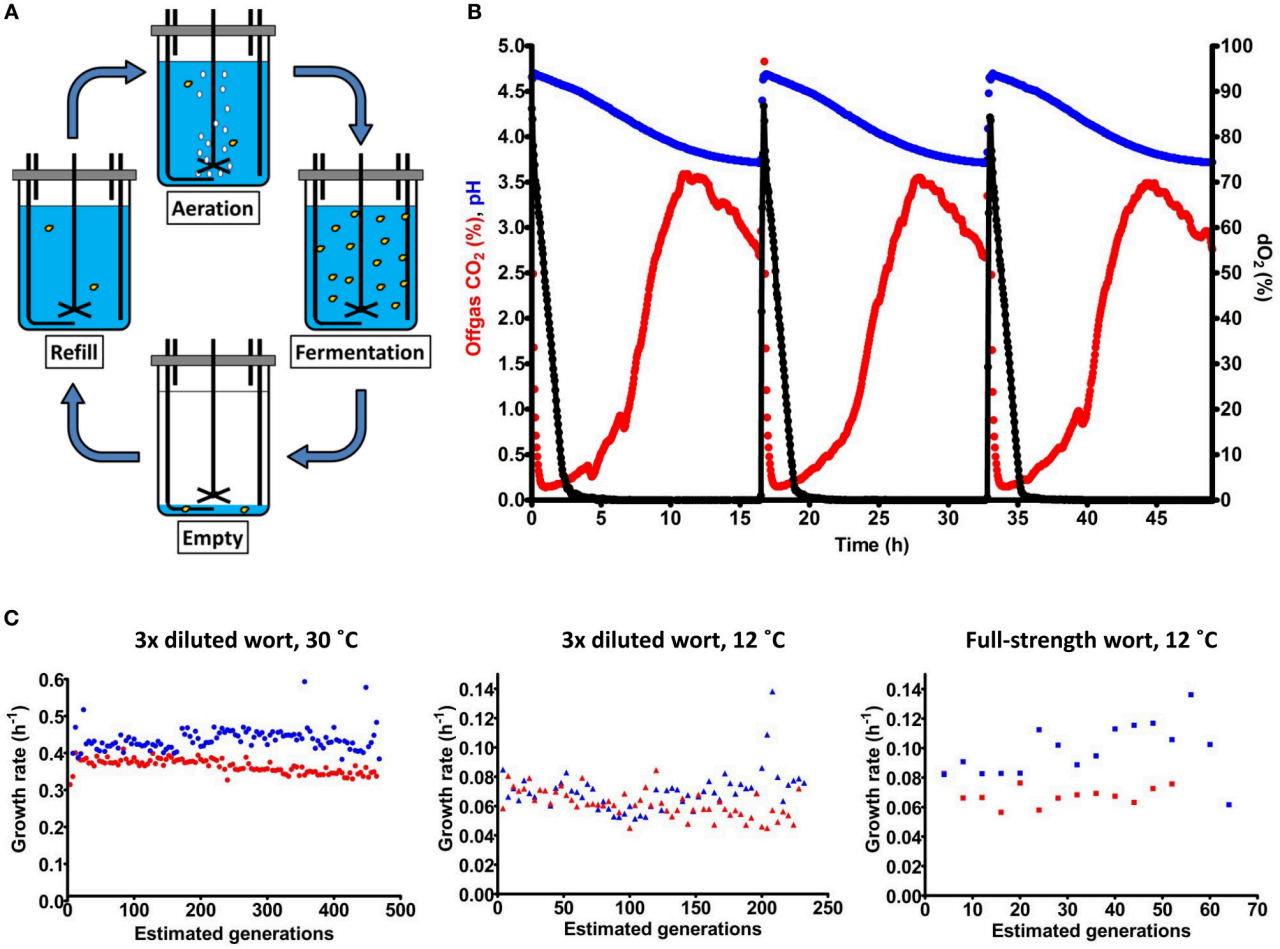
Laboratory evolution mimicking the domestication of lager-brewing yeast. The *S. cerevisiae* x *S. eubayanus* laboratory hybrid IMS0408 was grown in duplicate sequential batch bioreactors in three-fold diluted wort at 30°C (LG30.1 and LG30.2) and at 12°C (LG12.1 and LG12.2), and in full-strength wort at 12°C (HG12.1 and HG12.2). **(A)** Experimental design for simulated sequential lager-beer brewing cycles. Each cycle consisted of four phases: (i) (re)filling of the fermenter with fresh medium up to a total volume of 100 mL, (ii) aeration at 200 mL/min while stirring at 500 RPM, (iii) a batch fermentation phase without sparging or stirring, while flushing the bioreactor headspace with N_2_ to enable accurate analysis of CO_2_ production and (iv) removal of broth, leaving 7 mL to inoculate the next cycle. **(B)** Fermentation profiles of three consecutive cycles from experiment LG30.1, performed at 30°C in three-fold diluted wort. Percentage of CO_2_ in the off gas, culture pH and dissolved oxygen (dO_2_) concentration are indicated by red, blue and black symbols, respectively. Due to the lack of stirring and sparging, CO_2_ was slowly released by the medium; emptying of the reactor was initiated when the offgas CO_2_ concentration dropped to 70% of its initial value as off-line analyses indicated that, at this point, all fermentable sugars had been consumed **(C)** Specific growth estimated from CO_2_ production profiles during each cycle of the evolution lines. LG30.1 (blue circles) and LG30.2 (red circles) were grown on three-fold diluted wort at 30°C; LG12.1 (blue triangles) and LG12.2 (red triangles) were grown on three-fold diluted wort at 12°C. HG12.1 (blue squares) and HG12.2 (red squares) were evolved in full-strength wort at 12°C. Since lack of sparging and stirring precluded exact estimates of specific growth rates, the calculated values should be taken as indicative.

Industrial lager brewing involves batch cultivation of *S. pastorianus* on wort, an extract from malted barley, at temperatures between 7 and 15°C. After a brief initial aeration phase to enable oxygen-dependent biosynthesis of unsaturated fatty acids and sterols (Andreasen and Stier, 1953; Gibson et al., 2007), brewing fermentations are not aerated or stirred, leading to anaerobic conditions during the main fermentation (Briggs et al., 2004). To simulate domestication under industrial lager-brewing conditions, a laboratory evolution regime was designed in which the laboratory-made *S. cerevisiae* x *S. eubayanus* hybrid IMS0408 was grown at 12°C in sequential batch bioreactors on industrial wort. As in industrial brewing, each cultivation cycle was preceded by an aeration phase, after which cultures were incubated without sparging or stirring until a decline of the CO_2_ production indicated a cessation of sugar consumption. The bioreactors were then partially emptied, leaving 7% of the culture volume as inoculum for the next aeration and fermentation cycle, which was initiated by refilling the reactor with sterile wort (Figures 1A,B). To mimic the low sugar concentrations during early domestication of *S. pastorianus* (Meussdoerffer, 2009), parallel duplicate experiments at 12°C were not only performed with full-strength 17 °Plato wort (“High Gravity,” experiments HG12.1 and HG12.2), but also with three-fold diluted wort (“Low Gravity,” experiments LG12.1 and LG12.2). To enable a larger number of generations during 4 months of operation, additional duplicate experiments on three-fold diluted wort were performed at 30°C (LG30.1 and LG30.2).

Concentration of the wort and temperature strongly affected the length of the fermentation cycles, which was 17 h for LG30.1 and LG30.2, 93 h for LG12.1 and LG12.2 and 205 h for HG12.1 and HG12.2. Experiments LG30.1 and LG30.2 involved 117 and 118 batch cycles, respectively, LG12.1 and LG12.2 covered 58 and 57 cycles, respectively, and HG12.1 and HG12.2 covered 13 and 16 cycles, respectively. At the inoculum size of 7% of the total culture volume, each cycle corresponded to ~4 generations. Specific growth rates, estimated from CO_2_ production rates during the exponential growth phase of the batch cycles, were not significantly different during the first and the last five cycles of each experiment (Student's *t*-test, *p* > 0.05). Average specific growth rates were 0.35 ± 0.02 h^−1^ for LG30.1, 0.42 ± 0.03 h^−1^ for LG30.2, 0.070 ± 0.013 h^−1^ for LG12.1, 0.062 ± 0.009 h^−1^ for LG12.2, 0.068 ± 0.007 h^−1^ for HG12.1 and 0.098 ± 0.018 h^−1^ for HG12.2. While the initial specific growth rate was clearly higher at 30°C than at 12°C, initial growth rates on diluted and full-strength wort were not significantly different. However, CO_2_ production from sugars continued much longer in full-strength wort. During brewing fermentation, depletion of nitrogen sources and oxygen limit biomass formation. Complete sugar conversion therefore depends on growth-independent alcoholic fermentation which, apparently, was much slower in cultures grown on full-strength wort. At the end of each evolution experiment, culture samples were streaked on YPD agar and 5 single colonies were isolated for each culture. For experiments LG12.1 and LG12.2, isolates were also made from intermediate samples after 29 cycles. Evolution lines LG30.2 and LG12.2 developed flocculence and isolates from these lines had two distinct colony morphologies: about half of the colonies were elevated and conically-shaped, while the other colonies shared the flat morphology of IMS0408 (Figure 3A). For each of these lines, five random colonies of each morphology were selected.

### Prolonged Growth Under Simulated Brewing Conditions Did Not Cause Large Ploidy Changes

Six independent sequential batch fermentation experiments under simulated brewing conditions, covering 52 to 468 generations, yielded 55 isolates. Staining with the DNA-binding fluorescent dye SYTOX Green and flow cytometry indicated genome sizes of the isolates between 17.6 and 23.5 Mbp (Supplementary Table 3). These values did not differ significantly from the 21.3 ± 1.9 Mbp genome size measured for the parental laboratory hybrid IMS0408 and therefore indicated the absence of large changes in genome content such as whole-genome duplications. For a detailed genotypic analysis, the genomes of the 55 isolates were sequenced using 150 bp pair-end reads with 101- to 189-fold coverage. A high quality-reference genome was constructed by combining the chromosome-level contigs from assemblies of CEN.PK113-7D and CBS 12357 generated with nanopore technology, including mitochondrial genome sequences (Salazar et al., 2017; Brickwedde et al., 2018).

Copy number analysis revealed whole-chromosome aneuploidies in only 5/55 isolates (Table 2). Relative to strain IMS0408, the total chromosome number of the isolates had not changed by more than one. Isolate IMS0556 (LG30.1) had gained a copy of *Sc*CHRVIII, IMS0560 (LG30.1) had gained a copy of *Se*CHRX, IMS0565 (LG30.2) had lost *Sc*CHRXIV and gained a copy of *Se*CHRXIV, IMS0595 (LG12.1) had gained a copy of *Se*CHRVIII and IMS0606 (LG12.2) had lost a copy of *Se*CHRVIII.

**Table 2:** Overview of phenotypic and genotypic changes in isolates obtained after laboratory evolution of the allodiploid laboratory hybrid IMS0408 under simulated lager-brewing conditions.

**Experiment**	**GEN#^**a**^**	**Isolate**	**M^**b**^**	**F^**c**^**	**R^**d**^**	**M$M_{DNA}^{e}$**	**Aneuploidy^**f**^, ^**g**^**	**Segmental aneuploidy and loss of heterozygosity^**f**^^,^^**g**^**	**SNPs^**f**^^,^^**h**^**	**INDELs^**f**^^,^^**h**^^,^^**i**^**
Unevolved		IMS0408	+	–	+					
LG12.1 (3x diluted wort, 12°C)	116	IMS0538	+	–	+					
		IMS0539	+	–	+					
		IMS0540	+	–	+				*SeHDA2*^A1651T^	*SeMED2*^462+3N^
		IMS0541	+	–	–	ρ^0^				
		IMS0542	+	–	+				*ScIQG1*^A2069C^	*SeKEX1*^1875−6N^
		IMS0543	+	+	+			Δ*Sc*(YKL032C-YKL054C)		
		IMS0544	+	+	+			Δ*Sc*(YKL032C-YKL054C), Δ*Sc*::*Se*(YLR154C-YLR_end_)	*SeSAC1*^G1093C^	
		IMS0545	+	+	+			Δ*Sc*(YKL032C-YKL054C)		*SeNAF1*^1404−30N, 1436−30N^
		IMS0546	+	+	–	ρ^−^		Δ*Sc*(YKL032C-YKL054C)		
		IMS0547	+	+	+			Δ*Sc*(YKL032C-YKL054C)		
	232	IMS0594	+	–	+					*SeELA1*^230+343N^, *SeIRA2*^1402−1N^, ScFLO9*
		IMS0595	+	–	+		2x*Se*(CHRVIII)	Δ*Se*::*Sc*(YOL_end_-YOL072W)		
		IMS0596	+	–	+				*SeIRA2*^C1376A^, *Sc*YER188W^T28A^	
		IMS0597	+	–	+			Δ*Se*::*Sc*(YOL_end_-YOL057W)	*SeNUP1*^C1205T^, *ScPDC2*^G372A^	
		IMS0598	+	–	+				*SeSRT1*^C359T^	*SeASG1*^2488+3N^
		IMS0599	+	+	+			Δ*Sc*(YKL032C-YKL054C), Δ*Se*::*Sc*(YLR154C-YLR_end_)		
		IMS0600	+	–	+			Δ*Se*::*Sc*(YOL_end_-YOL075W)	*SeMSR1*^A853C^	
		IMS0601	+	+	–	ρ^−^		Δ*Sc*(YKL032C-YKL054C)		
		IMS0602	+	+	+			Δ*Sc*(YKL032C-YKL054C)		
		IMS0603	+	–	+			Δ*Se*::*Sc*(YNL_end_-YNL123W), Δ*Se*::*Sc*(YOL_end_-YOL013C),Δ*Sc*::*Se*(YOL013C-YOL006C)		
LG12.2 (3x diluted wort, 12°C)	116	IMS0548	+	–	+			Δ*Se*::*Sc*(YKL_end_-YKL057C), Δ*Sc*::*Se*(YKL057C-YKR_end_)		
		IMS0549	+	–	–	ρ^−^				
		IMS0550	+	–	–	ρ^−^				
		IMS0551	+	–	–	ρ^−^		Δ*Sc*(YCL_end_-YCL067C), Δ*Sc*(YCR039C-YCR_end_)		
		IMS0552	+	–	–	ρ^−^		Δ*Sc*::*Se*(YHL_end_-YHL023C)		
	228	IMS0604	+	–	+			Δ*Sc*(YKL032C-YKL054C), Δ*Sc*::*Se*(YLR305C-YLR_end_)	*SeBET2*^G550A^	
		IMS0605	+	–	+			Δ*Sc*(YKL032C-YKL054C)		
		IMS0606	+	–	–	ρ^−^	Δ*Se*(CHRVIII)	Δ*Sc*(YKL032C-YKL054C)	*ScLRG1*^C2277G^	
		IMS0607	+	–	+			Δ*Sc*(YKL032C-YKL054C)		SeFLO11*
		IMS0608	+	–	-	ρ^−^		Δ*Sc*(YKL032C-YKL054C)		
LG30.1 (3x diluted wort, 30°C)	464	IMS0553	+	–	+					*SeNHX1*^1622+3N^
		IMS0554	-	–	+			Δ*Sc*::*Se*(YGR282C-YGR_end_)		ScCIS3*
		IMS0555	+	–	+				*SeBAT1*^G1073A^, *ScMAL23*^G422A^	
		IMS0556	+	–	+		2x*Sc*(CHRVIII)		*SeGMC1*^G1579A^, *ScSFL1*^T605A^	
		IMS0557	–	–	+				*ScMAL11*^G88T, A98G^, *ScMDL2*^C1451A^	*ScMAL11*^93−1N^*, Se*YNL247W^2062−1N^
		IMS0558	-	+	+			Δ*Sc*(YDR261C-YDR211W), Δ*Sc*::*Se*(YGR218C-YGR_end_)	*ScSFL1*^T605A^	*SeSFL1*^96+1N^
		IMS0559	+	+	+			Δ*Se*::*Sc*(YOR133W-YOR_end_)	*ScSFL1*^T605A^	
		IMS0560	+	+	+		2x*Se*(CHRX)	Δ*Sc*(YDR261C-YDR211W), Δ*Se*::*Sc*(YOR063W-YOR_end_)	*ScSFL1*^T605A^	
		IMS0561	+	+	+			Δ*Se*::*Sc*(YBR275C-YBR_end_), Δ*Se*::*Sc*(YOR133W-YOR_end_)	*ScSFL1*^T605A^	
		IMS0562	+	+	+			Δ*Sc*::*Se*(YNL061C-YNL055C), Δ*Se*::*Sc*(YOR133W-YOR_end_)	*ScSFL1*^T605A^	
LG30.2 (3x diluted wort, 30°C)	468	IMS0563	-	–	+			Δ*Sc*(YGR279C-YGR_end_)::*Se*(YMR305C-YMR_end_)	*SeROG3*^G191A^, *SeMSS51*^C448T^	*ScRTP1*^874−64N^
		IMS0564	+	–	+				*SeIZH3*^A526G^, *ScCST6*^C807A^	
		IMS0565	-	–	+		Δ*Sc*::*Se*(CHRXIV)		*ScMAL11*^A1G^	
		IMS0566	+	–	–	ρ^−^			*ScERG6*^C413T^, *ScBUL1*^G2110A^	*Se*YBR238C^315−36N^
		IMS0567	–	–	–	ρ^−^		Δ*Sc*::*Se*(YDR051C-YDR_end_), Δ*Sc*::*Se*(YGR271W-YGR_end_)		*ScULP2*^1469+1N^
HG12.1 (Full-strength wort, 12°C)	52	IMS0609	+	–	+					
		IMS0610	+	–	-	ρ^−^			*Sc*YBR259W^C833A^	
		IMS0611	+	–	-	ρ^−^			*ScFMP52*^G406C^	
		IMS0612	+	–	+					
		IMS0613	+	–	+				*SeGIC2*^C344G,^	*SeGCN2*^2274+18N, 2239+18*N,*^ *SeTRA1*^4421−25N^
HG12.2 (Full-strength wort, 12°C)	64	IMS0614	+	–	+			Δ*Se*(YAR050W-YARend)::*Se*(YALend-YAL063C)	*SeSFL1*^C1390T^	
		IMS0615	+	–	+				*ScCAC2*^A994T^	
		IMS0616	+	–	–	ρ^−^			*ScATG1*^A434C^	
		IMS0617	+	–	+				*SeSFL1*^C1390T^	
		IMS0618	+	–	–	ρ^−^			*ScALR1*^G1645T^	

*A complete list of mutations is provided in Supplementary Data File 1*.

a *Estimated number of generations prior to isolation*.

b *Ability to utilize the sugar maltotriose*.

c *Flocculation during growth on liquid medium*.

d *Respiratory capacity*.

e *Presence of the mitochondrial DNA. An empty field indicates presence of the S. eubayanus mitochondrial DNA, ρ^−^ indicates complete loss of the mitochondrial genome and ρ^0^ partial loss*.

f *Sc and Se indicate sequences on the S. cerevisiae and S. eubayanus subgenomes, respectively*.

g *“Δ” indicates deletion, “2x” indicates duplication and “::” indicates substitution*.

h *SNPs and INDELs are only indicated when they affected an ORF and were non synonymous*.

i *The number of nucleotides added (+N) or removed (–N) is indicated after the coordinate of the last unchanged nucleotide*.

FLO9 cer * a indicate coverage drops within these ORFs, for which the responsible mutation could not be exactly reconstructed.

Read alignments to mitochondrial genome sequences were absent from 14/55 isolates, while 1 isolate showed only a partial alignment, indicating complete (ρ^−^) or partial loss (ρ^0^) of the mitochondrial genome in 15/55 strains. Loss of respiratory competence was confirmed by the observation that these 15 isolates, in contrast to IMS0408 and isolates containing a full mitochondrial genome, were unable to grow on YP-ethanol (Supplementary Figure 1).

### Chromosomal Recombinations Frequently Caused Loss of Heterozygosity

**Figure 2:**
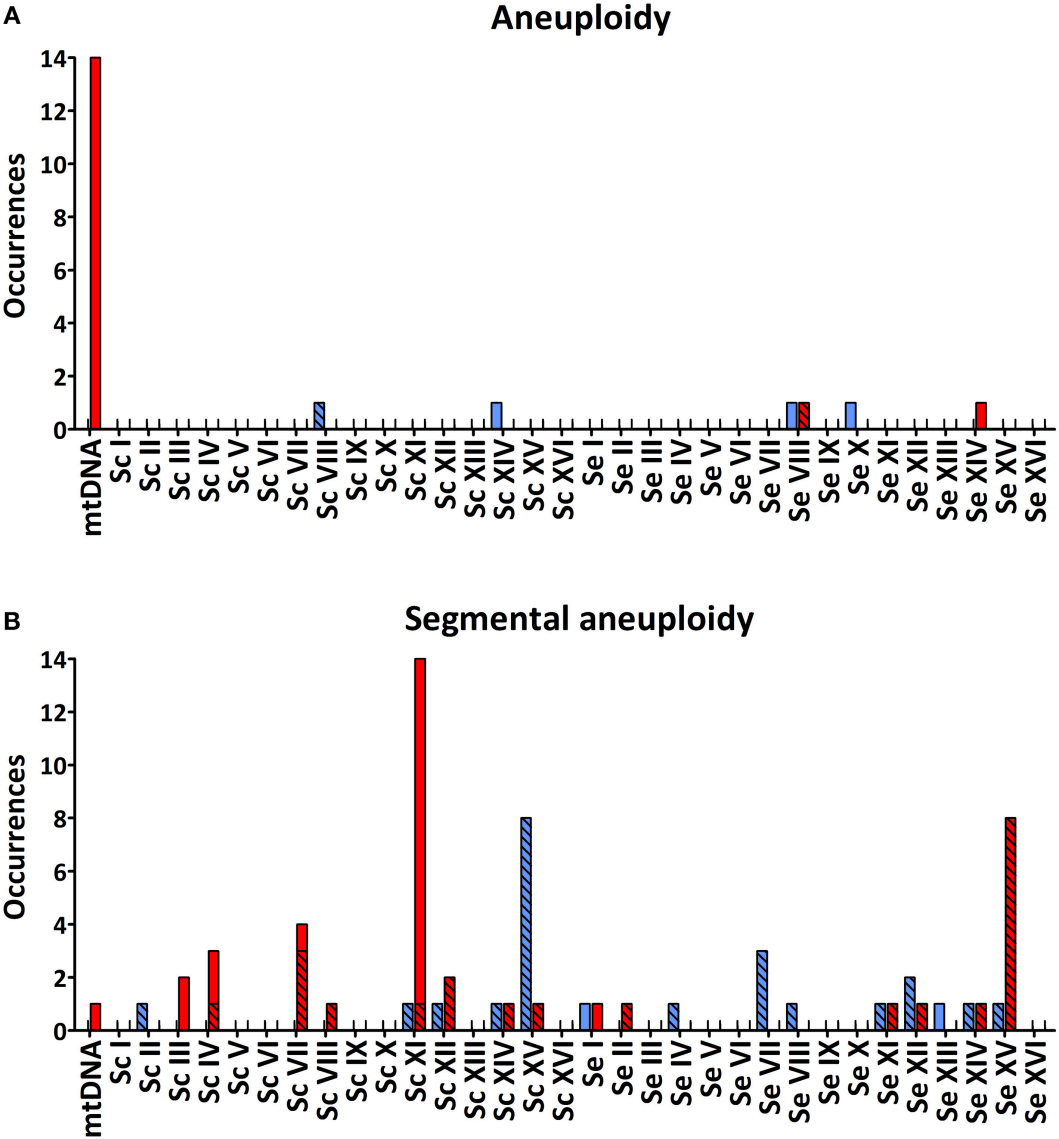
Total number of occurrences of whole-chromosome **(A)** and segmental **(B)** aneuploidy for each chromosome of IMS0408 among 55 isolates obtained after laboratory evolution under simulated lager fermentation conditions. For each chromosome, loss of genetic material is indicated in red and duplicated genetic material is indicated in blue. Loss or duplication of *S. cerevisiae* or *S. eubayanus* genetic material which was coupled with duplication or loss of the corresponding region of the other subgenome, is indicated by checked bars. *S. eubayanus* harbors two translocations relative to *S. cerevisiae:* between chromosomes II and IV, and between chromosomes VIII and XV. For simplicity, copy number affecting these regions were allocated based on the *S. cerevisiae* genome architecture.

Of the 55 evolved isolates, 29 displayed segmental copy number changes. In total, 20 of the 32 chromosomes in strain IMS0408 were affected in at least one isolate. Of the 55 evolved isolates, 24 showed chromosome segments with increased copy number and 41 showed chromosome segments with decreased copy number (Table 2, Figure 2). Seventeen internal recombinations resulting in deletions were observed: Δ*Sc*(YKL032C-YKL054C) occurred in 13 strains, Δ*Sc*(YDR261C-YDR211W) occurred in two strains, and Δ*Sc*(YCL_end_-YCL067C) and Δ*Sc*(YCR039C-YCR_end_) occurred together in one strain. The internal recombinations Δ*Sc*(YKL032C-YKL054C) and Δ*Sc*(YDR261C-YDR211W) both resulted in loss of the sequence between the recombination sites. The recombination occurred between *IXR1* and *DEF1* in Δ*Sc*(YKL032C-YKL054C) and between Ty-transposons for Δ*Sc*(YDR261C-YDR211W). Finally, the concurrent loss of *Sc*(YCL_end_-YCL067C) and *Sc*(YCR039C-YCR_end_) indicated loss of both ends of *Sc*CHRIII, including telomeres. This mutation is consistent with a previously-observed circularization of chromosome III by a recombination between the HMLALPHA2 (YCL067C) and MATALPHA2 (YCR039C) loci, leading to loss of both chromosome extremities (Newlon et al., 1991).

The remaining 24 chromosome-segment duplications and losses reflected inter-chromosomal recombinations: one chromosomal region was replaced by an additional copy of another chromosomal region by a non-conservative recombination. The recombinations Δ*Sc*(YGR279C-YGR_end_)::*Se*(YMR305C-YMR_end_) and Δ*Se*(YAR050W-YAR_end_)::*Se*(YAL_end_-YAL063C) occurred between highly similar genes; the paralogs *SCW4* and *SCW10*, and *FLO1* and *FLO9*, respectively. In the remaining 22 cases, recombination occurred between homologous genes of each subgenome. No copy-number conservative chromosome translocations were identified.

Of the 26 observed recombinations, 23 occurred inside ORFs, and thus resulted in chimeric genes (Table 3). The homology between ORFs involved in recombinations varied from < 70 to 100%, with a median homology of 82.41%. Chimeric ORFs were reconstructed by extracting reads from one locus affected by the recombination which were paired to the other locus affected by the recombination from the sequencing data, and using them for a local assembly. This approach allowed for identification of the recombination site at a resolution that, depending on sequence homology of the two ORFs, varied between 2 and 633 nucleotides. Due to length differences and relative INDELs between the original ORFs, recombined ORFs differed in length. However, all recombinations occurred in frame and no premature stop codons were introduced, suggesting that these chimeric ORFs might yield functional proteins.

**Table 3:** Overview of all recombinations observed in 55 isolates obtained after laboratory evolution of strain IMS0408 under simulated lager fermentation conditions.

**Recombination^**a**^^,^^**b**^**	**Affected isolates**	**Locus 1**			**Locus 2**			**Homology^**e**^**	**Length chimeric ORF (bp)**
		**Name^**a**^**	**Length^**c**^**	**Recombination^**d**^**	**Name^**a**^**	**Length^**c**^**	**Recombination^**d**^**		
Δ*Se*(YAR050W-YARend)::*Se*(YALend-YAL063C)	IMS0614	*SeFLO1*	5,517	711–734	*SeFLO9*	4752	712–735	79.23%	4,752
Δ*Se*::*Sc*(YBR275C-YBRend)	IMS0561	*ScRIF1*	5,751	562–572	*SeRIF1*	5,751	569–579	77.49%	5,745
Δ*Sc*::*Se*(YDR051C-YDRend)	IMS0567	*SeDET1*	1,005	426–434	*ScDET1*	1,005	427–435	84.36%	1,005
Δ*Sc*::*Se*(YGR218C-YGRend)	IMS0558	*ScCRM1*	3,251	2,626–2,636	*SeCRM1*	3,251	2,627–2,637	85.84%	3,251
Δ*Sc*::*Se*(YGR271W-YGRend)	IMS0567	*ScSLH1*	5,904	1,335–1,349	*SeSLH1*	5,901	1,336–1,350	82.41%	5,901
Δ*Sc*(YGR279C-YGRend)::*Se*(YMR305C-YMRend)	IMS0563	*SeSCW10*	1,146	892–899	*ScSCW4*	11,61	908–915	< 70%*	1,146
Δ*Sc*::*Se*(YGR282C-YGRend)	IMS0554	*SeBGL2*	942	115–125	*ScBGL2*	942	116–126	87.75%	942
Δ*Sc*::*Se*(YHLend-YHL023C)	IMS0552	*SeNPR3*	3,444	1,551–1,571	*ScNPR3*	3,441	1,561–1,581	78.80%	3,432
Δ*Se*::*Sc* (YKLend-YKL057C), Δ*Sc*::*Se*(YKL057C-YKRend)	IMS0548	*SeNUP120*	3,114	1,867–1,868	*ScNUP120*	3,114	1,868–1,869	80.14%	3,114
Δ*Sc*::*Se*(YLR154C-YLRend)	IMS0544	Ribosomal DNA	–	–	Ribosomal DNA	–	–	–	–
Δ*Se*::*Sc*(YLR154C-YLRend)	IMS0599	Ribosomal DNA	–	–	Ribosomal DNA	–	–	–	–
Δ*Sc*::*Se*(YLR305C-YLRend)	IMS0604	*SeSTT4*	5,703	5,013–5,018	*ScSTT4*	5,703	5,014–5,019	81.64%	5,703
Δ*Sc*::*Se*(YNL061C-YNL055C)	IMS0562	*ScNOP2*	1,857	1,386–1,391	SeNOP2	1,860	1,390–1,395	87.11%	1,857
		*ScPOR1*	852	399–401	*Se*POR1	852	400–402	85.82%	852
Δ*Se*::*Sc*(YNLend-YNL123W)	IMS0603	Sc*NMA111*	2,994	303–314	*SeNMA111*	2,994	304–315	83.61%	2,994
Δ*Sc*::*Se*(YOL013C-YOL006C)	IMS0603	*SeTOP1*	2,304	2,010–2,021	*ScTOP1*	2,311	2,017–2,028	82.64%	2,304
Δ*Se*::*Sc*(YOLend-YOL013C)	IMS0603	*SeHRD1*	1,644	312–329	*Sc*HRD1	1,656	313–330	82.82%	1,656
Δ*Se*::*Sc*(YOLend-YOL057W)	IMS0597	*Sc*YOL057W	3,015	99–116	*Se*YOL057W	2,133	100–117	< 70%*	2,133
Δ*Se*::*Sc*(YOLend-YOL072W)	IMS0595	*ScTHP1*	1,368	726–731	*SeTHP1*	1,374	733–738	79.07%	1,368
Δ*Se*::*Sc*(YOLend-YOL075W)	IMS0600	*SeYOL075C*	3,903	2,409–2,414	*Sc*YOL075C	3,885	2,392–2,397	81.85%	3,903
Δ*Se*::*Sc*(YOR063W-YORend)	IMS0560	*ScRPL3*	1,164	1,023–1,063	*SeRPL3*	1,164	1,024–1,064	94.59%	1,164
Δ*Se*::*Sc*(YOR133W-YORend)	IMS0559, IMS0561 and IMS0562	*ScEFT1*	2,529	246–311	*SeEFT1*	2,529	247–312	94.94%	2,529
Δ*Sc*(YCLend-YCL067C), Δ*Sc*(YCR039C-YCRend)	IMS0551	*Sc*HMLALPHA2	633	1–633	*Sc*MATALPHA2	633	1–633	100%	633
Δ*Sc*(YDR261C-YDR211W)	IMS0558 and IMS0560	TY-transposon	–	–	TY-transposon	–	–	–	–
Δ*Sc*(YKL032C-YKL054C)	IMS0543-547, IMS0599, IMS0601 and IMS0602	*ScIXR1*	1,794	944–955	*ScDEF1*	2,217	1,281–1,292	< 70%*	1,881
Δ*Sc*(YKL032C-YKL054C)	IMS0604-608	*ScIXR1*	1,794	332–341	*ScDEF1*	2,217	1,272–1,281	< 70%*	1,278

a *Sc and Se indicate sequences on the S. cerevisiae and S. eubayanus subgenomes, respectively*.

b *“Δ” indicates deletion and “::” indicates substitution*.

c *Length of the affected ORF in bp*.

d *Nucleotide coordinates of the locus at which recombination could have occurred. Due to the high homology of Locus 1 and Locus 2, they shared identical sequence stretches within which the recombination could have occurred. Recombination sites are therefore indicated by a range of nucleotide coordinates within the ORFs*.

e *Homology between Locus 1 and Locus 2 as determined by sequence alignment*.

### *IRA2, SFL1*, and *MAL11* Are Mutated in Multiple Evolved Isolates

A total of 76 SNPs and 43 INDELs were identified in the genomes of the 55 isolates (Supplementary Data Files 1A,B), of which 38 SNPs and 17 INDELs occurred in ORFs and were non-synonymous (Table 2). Gene ontology analysis of all genes affected by non-synonymous SNPs or INDELs did not yield a significant enrichment in specific biological processes, molecular functions or cellular components. However, the genes *IRA2, SFL1*, and *MAL11* were affected in more than one strain. *IRA2* encodes a RAS GTPase-activating protein, which is disrupted in many *S. cerevisiae* genomes from the CEN.PK strain family (Tanaka et al., 1990, 1991; Nijkamp et al., 2012). In strain IMS0408, the *ScIRA2* was indeed disrupted while the *SeIRA2* ORF was intact. However, *SeIRA2* was mutated in 6/10 isolates from LG12.1 after 232 generations. *SeIRA2* had a frameshift in IMS0594, a premature stop codon in IMS0596 and was completely lost in four isolates due to different loss of heterozygosities: Δ*Se*::*Sc*(YOL_end_-YOL072W) in IMS0595, Δ*Se*::*Sc*(YOL_end_-YOL057W) in IMS0597, Δ*Se*::*Sc*(YOL_end_-YOL075W) in IMS0600 and ΔSe::Sc(YOL_end_-YOL013C) in IMS0603.

*SFL1* encodes a transcriptional repressor of flocculation genes, which was present both on *Sc*CHRXV and *Se*CHRVIII (Atsushi et al., 1989). *ScSFL1* was mutated in 6/10 isolates from LG30.1 after 464 generations, which harbored a non-conservative substitution at the 605th nucleotide, affecting its DNA binding domain (Atsushi et al., 1989). *SeSFL1* had a frameshift in IMS0558 (LG30.1), a single nucleotide substitution in IMS0614 and IMS0617 (HG12.2) and was completely lost in four isolates of LG30.1 due to two losses of heterozygosity: Δ*Se*::*Sc*(YOR133W-YOR_end_) in IMS0559, IMS0561 and IMS0562, and Δ*Se*::*Sc*(YOR063W-YOR_end_) in IMS0560 (Table 2).

*ScMAL11*, also referred to as *AGT1*, encodes the only maltose transporter of the *MAL* gene family which enables efficient uptake of maltotriose in IMS0408 (Alves et al., 2008). Sc*MAL11* is located on the right arm of *Sc*CHRVII and is absent in the *S. eubayanus* subgenome of IMS0408, which has no other maltotriose transporters (Hebly et al., 2015; Brickwedde et al., 2018). *ScMAL11* had a frameshift in IMS0557 (LG30.1) and lost its start codon in IMS0565 (LG30.2) (Table 2). In addition, *ScMAL11* was completely lost due to three different losses of heterozygosity: Δ*Sc*::*Se*(YGR282C-YGR_end_) in IMS0554 (LG30.1), Δ*Sc*::*Se*(YGR218C-YGR_end_) in IMS0558 (LG30.1) and Δ*Sc*::*Se*(YGR271W-YGR_end_) in IMS0567 (LG30.2), and due to the non-conservative recombination Δ*Sc*(YGR279C-YGR_end_)::*Se*(YMR305C-YMR_end_) in IMS0563 (LG30.2).

### Mutations in *SFL1* Cause Emergence of Calcium-Dependent Flocculation

**Figure 3:**
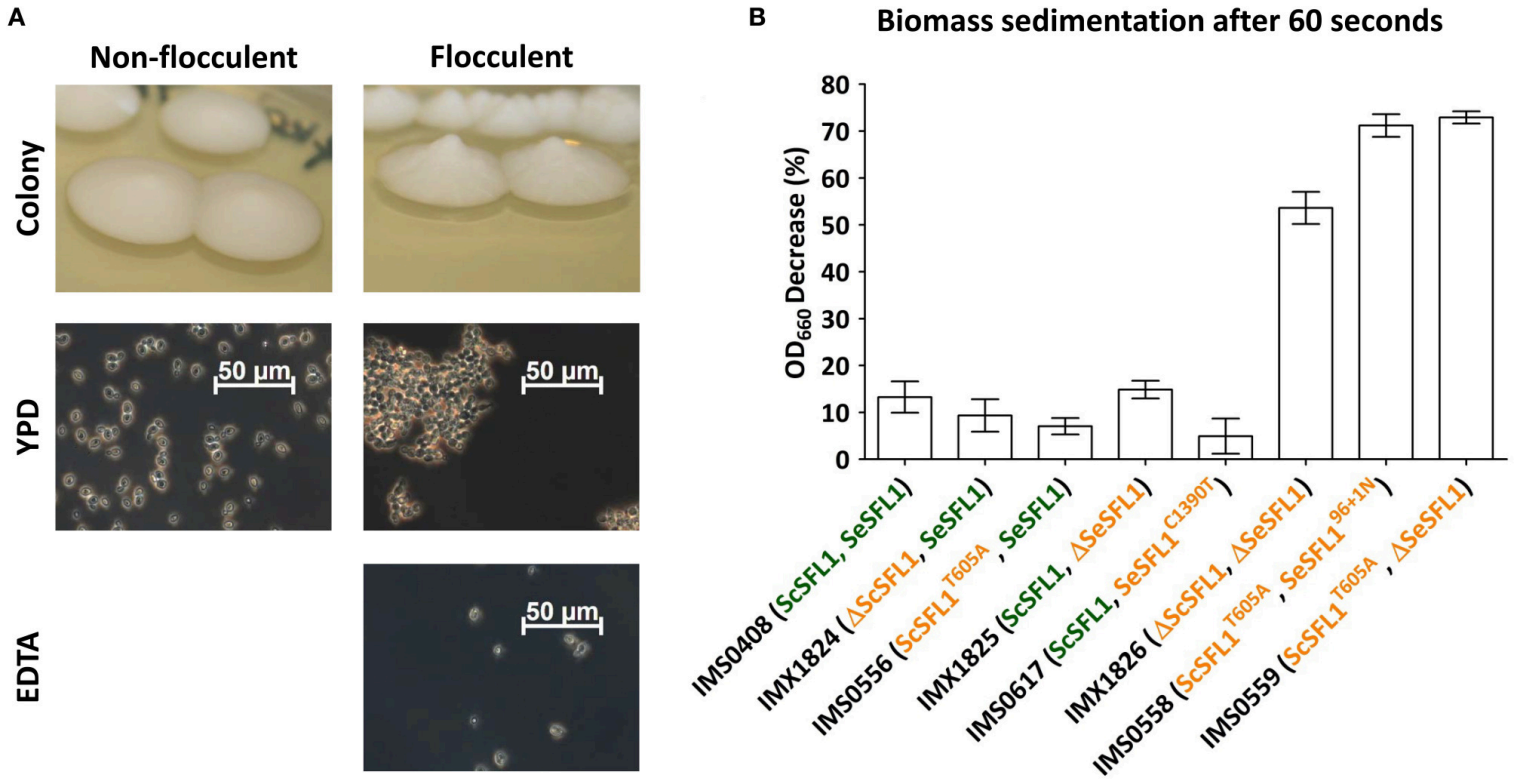
Mutations in *ScSFL1* and *SeSFL1*correlate with flocculation in evolved isolates and reverse engineered strains. **(A)** Colony morphology and phase-contrast microscopy images (100x) of YPD-grown cell suspensions of the non-evolved, non-flocculent strain (IMS0408), and of a typical flocculent evolved isolate (IMS0558). Resuspension in 50 mM EDTA (pH 7.0) eliminated flocculation. **(B)** Biomass sedimentation of evolved isolates and engineered strains with mutations in *SeSFL1* and/or *ScSFL1*. Triplicate cultures of all strains were grown on YPD and sedimentation was measured as the decrease in OD_660_ right underneath the meniscus of a stationary cell suspensions 60 s after the suspension had been vortexed.

Eight of the 55 evolved isolates showed SNPs, INDELs or loss of heterozygosity in the flocculation inhibitor gene *SFL1*. In isolates IMS0558, IMS0559, IMS0560, IMS0561, and IMS0562 from LG30.1, both *SeSFL1* and *ScSFL1* were mutated, while in isolate IMS0556 from LG30.1 only *ScSFL1* was mutated and in isolates IMS0614 and IMS0617 from HG12.2, only *SeSFL1* was mutated. Evolved isolates that carried mutations in both *ScSFL1* and *SeSFL1* formed elevated conically-shaped colonies on YPD agar, while strain IMS0408 and evolved isolates with either an intact *SeSFL1* or *ScSFL1* did not (Figure 3A). Strains with mutations in both *ScSFL1* and *SeSFL1* also showed rapid sedimentation in micro-aerobic cultures on wort, which was not observed for the other evolved isolates or for strain IMS0408 (Figure 3B).

### Mutations in *ScMAL11* Cause Loss of Maltotriose Utilization

All evolved isolates and the unevolved hybrid IMS0408 were grown under brewing conditions at the temperature and wort gravity used during their evolution in micro-aerobic 100 mL bottles to assess their brewing performance. Besides differences in flocculation behavior described above, only 6 isolates evolved on diluted wort at 30°C displayed significantly different brewing performances relative to IMS0408. These six strains all harbored mutations to the *ScMAL11* gene, which encodes the sole maltotriose transporter in strain IMS0408. The unevolved IMS0408 consumed 100 ± 0% of the maltotriose in diluted wort, and evolved strains with an intact *ScMAL11* genes consumed 98 ± 3%. In contrast, strains IMS0554, IMS0557, IMS0558, IMS0563, IMS0565, and IMS0567, which all harbored mutations in *ScMAL11*, did not show any maltotriose consumption; instead, the concentration increased by 14 ± 3% on average, presumably due to water evaporation. To test if the mutations affecting *ScMAL11* were responsible for the loss of maltotriose utilization, *ScMAL11* was deleted in strain IMS0408 using CRISPR-Cas9 gene editing, resulting in strain IMX1698 (mVenus::Δ*Scmal11*). Under the same conditions used to evaluate maltotriose utilization by the evolved strains, strain IMS0408 consumed 97 ± 5% of the maltotriose while strain IMX1698 only consumed 1 ± 0% of the maltotriose. These results confirmed that loss of *ScMAL11* function was responsible for loss of maltotriose utilization.

## Discussion

**Figure 4:**
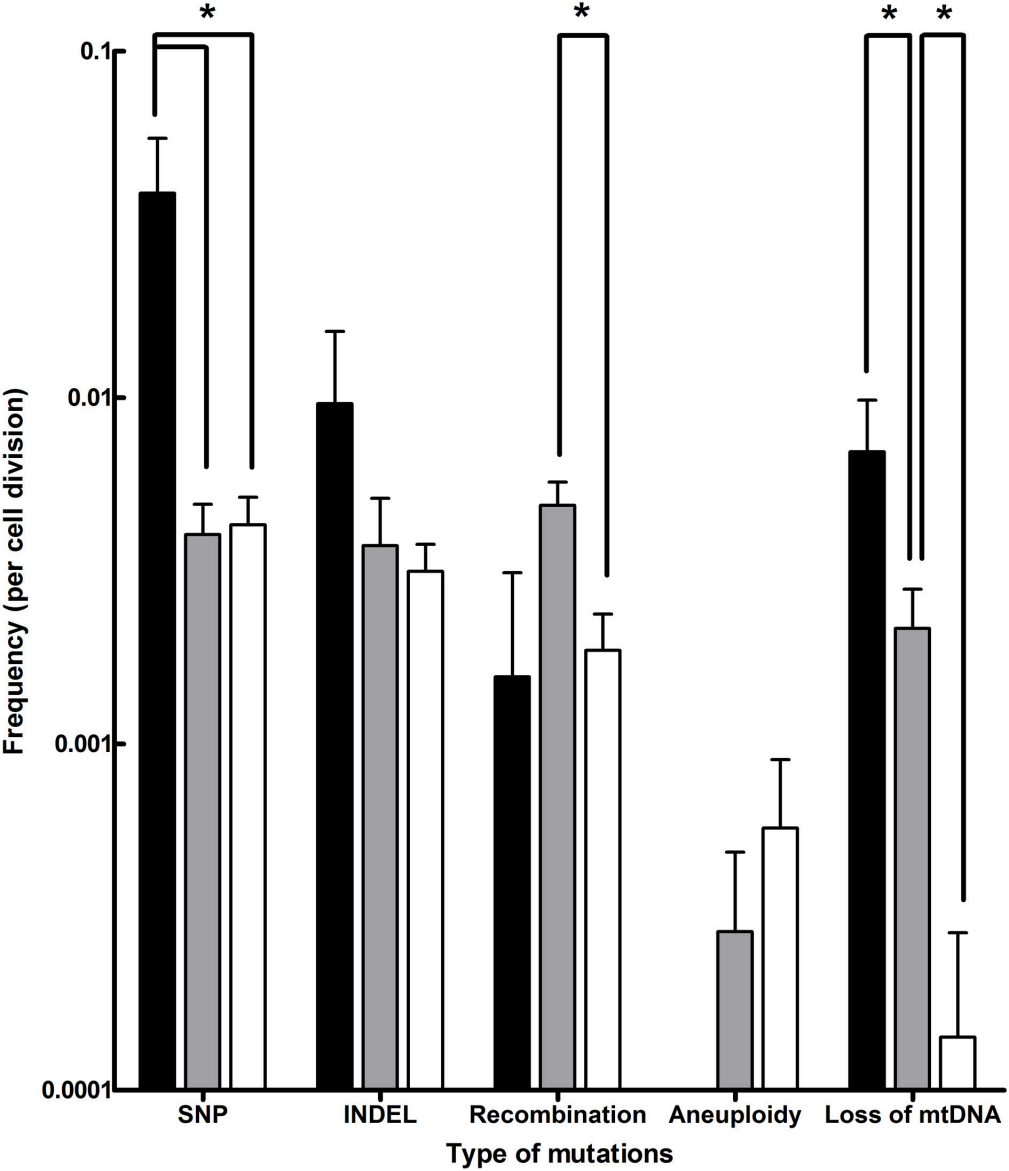
Frequencies of different types of mutations observed in evolved isolates obtained after laboratory evolution of strain IMS0408 under simulated lager fermentation conditions. The mutations identified in all 55 isolates evolved under brewing conditions were classified by type, and the frequency of mutation per cell division was calculated for each isolate based on its estimated number of generations of growth under simulated brewing conditions. Average frequencies of mutation types and standard deviations are shown for isolates evolved on full-strength wort at 12°C (black), on three-fold diluted wort at 12°C (gray) and on threefold diluted wort at 30°C (white). The frequencies are shown on a logarithmic scale, and *p*-values were determined using Student's *t*-test. * indicates Significant differences (*p*-value < 0.05).

Evolution of the laboratory *S. cerevisiae* x *S. eubayanus* IMS0408 under simulated lager-brewing conditions yielded a wide array of mutations, including SNPs, INDELs, chromosomal recombinations, aneuploidy and loss of mitochondrial DNA (Table 2, Additional Data File 1). SNPs were the most common type of mutation, with frequencies ranging between 0.004 and 0.039 per division (Figure 4). Based on a genome size of 24.2 Mbp, the rate at which single-nucleotide mutations occurred was between 1.7·10^−10^ and 1.6·10^−9^ per nucleotide per cell division, which is similar to a rate of 3.3·10^−10^ per site per cell division reported for *S. cerevisiae* (Lynch et al., 2008). At a frequency of 0.003 and 0.010 per cell division, INDELs occurred up to 4.1-fold less frequently than SNPs, in accordance with their two-fold lower occurrence in *S. cerevisiae* (Lang and Murray, 2008). The higher incidence of both SNPs and INDELs in isolates evolved in full-strength wort (Figure 4) may be related to the higher concentrations of ethanol, a known mutagen, in these cultures (Voordeckers et al., 2015). The rate of loss of mitochondrial DNA varied between 0.0001 and 0.007 per division (Figure 4), and was negatively correlated with the number of generations of selective growth, indicating loss of mitochondrial DNA is selected against. The percentage of respiratory deficient isolates, between 13 and 40% for all evolutions, is consistent with observations during laboratory evolution under oxygen limitation, and has been associated with increased ethanol tolerance (Ibeas and Jimenez, 1997; Taylor et al., 2002). However, loss of reparative capacity is highly undesirable for lager brewing as it impedes biomass propagation (Gibson et al., 2008).

The frequency of chromosomal recombinations was estimated between 0.002 and 0.005 per division (Figure 4), which is similar to frequencies reported for *S. cerevisiae* (Dunham et al., 2002). The observed recombinations were not reciprocal translocations. Instead, in all cases, genetic material was lost due to internal deletions, or genetic material from one chromosome was replaced by an additional copy of genetic material from another chromosome. This abundant loss of heterozygosity is consistent with the evolutionary history of *S. cerevisiae* (Magwene et al., 2011) and with previously observed loss of genetic material in hybrids (Sipiczki, 2008; Peris et al., 2017; Smukowski Heil et al., 2017, 2018). Under selective conditions, regions from one subgenome can be preferentially affected by loss of heterozygosity due to the fitness effects of genes they harbor. Due to its irreversibility, loss of heterozygosity is a determining mechanism for the evolution of hybrids during domestication (Pérez Través et al., 2014). For example, in an *S. cerevisiae* x *S. uvarum* hybrid, the chromosomal region harboring *ScPHO84* was preferentially retained at 30°C, while it was lost at the expense of its *S. uvarum* homolog at 15°C (Smukowski Heil et al., 2017, 2018). Similarly, the superior growth of *S. cerevisiae* at 30°C relative to *S. paradoxus* could be linked to *S. cerevisiae* alleles of eight genes by investigating the effect of loss of heterozygosity in a laboratory hybrid (Weiss et al., 2018). Moreover, the deletion of *S. cerevisiae* alleles in *S. uvarum* × *S. cerevisiae* had strong and varying impacts on fitness under glucose-, sulfate- and phosphate-limitation (Lancaster et al., 2018). Overall, loss of heterozygosity is an irreversible process which enables rapid adaptation of hybrid genomes to the selective pressure of their growth environment. In the present study, loss of heterozygosity notably affected *ScMAL11* and *SeSFL1*, contributing to the acquired flocculation and loss of maltotriose utilization phenotypes. Other observed losses of heterozygosity may be due to genetic drift, or they may yield a selective advantage which we did not identify. Loss of heterozygosity may be advantageous by removing unfavorable dominant alleles, by enabling the expression of favorable recessive alleles, or by resolving redundancy, cross-talk and possible incompatibility between alleles and genes of both subgenomes (Piatkowska et al., 2013; Gibson and Liti, 2015).

Whole-chromosome aneuploidy was the rarest type of mutation, occurring only between 0 and 0.0003 times per division (Figure 4). The emergence of single chromosome aneuploidy in 9% of the isolates after laboratory evolution is similar to observations during laboratory evolution of *S. cerevisiae* (Yona et al., 2012; Voordeckers et al., 2015; González-Ramos et al., 2016; Gorter de Vries et al., 2017b). However, the observed small extent of aneuploidy starkly contrasts with the massive aneuploidy of *S. pastorianus* brewing strains (Van Den Broek et al., 2015; Okuno et al., 2016). These differences might of course be attributed to the difference in time scale between 4 months of laboratory evolution and several centuries of domestication. However, they might also be due to differences between the *S. cerevisiae* × *S. eubayanus* IMS0408 evolved in this study and the ancestral *S. pastorianus* hybrid. Firstly, IMS0408 was obtained by crossing a haploid *S. cerevisiae* strain with a haploid *S. eubayanus* spore (Hebly et al., 2015). In contrast, the progenitor of brewing strains of *S. pastorianus* may have resulted from a cross of higher ploidy strains (Okuno et al., 2016). Since higher ploidy leads to higher chromosome missegregation rates, they accumulate chromosome copy number changes faster (Storchova, 2014). In addition, the higher initial ploidy leads to a smaller relative increase of genetic material when an additional copy is gained, which may “buffer” deleterious effects of further changes in ploidy (Torres et al., 2007). Moreover, this laboratory-made hybrid was constructed by crossing a laboratory *S. cerevisiae* strain with the first discovered *S. eubayanus* strain, which may differ considerably from the genetic background of the initial *S. pastorianus* hybrid (Okuno et al., 2016). Therefore, the ancestral *S. pastorianus* hybrid may have had, or have acquired, mutations that stimulate extensive aneuploidy, such as mutations increasing the rate of chromosome missegregation or mutations increasing the tolerance against aneuploidy associated stresses (Torres et al., 2010). Finally, aneuploidy may have emerged in *S. pastorianus* by an (aborted) sporulation event, as such events can cause uneven chromosome segregation even in non-hybrid polyploid yeasts (Kim et al., 2017). Regardless of the origin of the extensive aneuploidy of *S. pastorianus*, our results show that euploid *S. cerevisiae* × *S. eubayanus* hybrids are not by definition prone to extensive aneuploidy under brewing-related experimental conditions. For industrial applications, the relative genetic stability of newly generated *Saccharomyces* hybrid strains reduces the chance of strain deterioration during the many generations involved in large-scale fermentation and/or biomass recycling (Krogerus et al., 2017). Due to the industry practice of limiting yeast biomass re-pitching, the genetic and phenotypic stability observed during 52 generations in 17°P wort at 12°C is sufficient to warrant sufficient strain stability for lager brewing applications.

In addition to showing extensive aneuploidy, *S. pastorianus* strains harbor numerous chromosomal recombinations. During the laboratory evolution experiments, two types of recombinations were observed: (i) intrachromosomal recombinations resulting in loss of chromosome segments, and (ii) interchromosomal recombinations resulting in loss of one chromosome segment and replacement by an additional copy of a segment from another chromosome, resulting in loss of heterozygosity. While in *S. cerevisiae*, chromosomal recombinations predominantly occur in repetitive regions of the genome (Dunham et al., 2002; Fontdevila, 2005), here 88% of the observed recombinations occurred within ORFs. The average homology of the recombined ORFs did not exceed the average 85% homology of ORFs in the *S. cerevisiae* and *S. eubayanus* subgenomes. Instead, the high rate of recombinations at ORFs could reflect a correlation between transcriptional activity and recombination (Thomas and Rothstein, 1989). In all cases, the reading frames were conserved, resulting in chimeric ORFs which could encode functional chimeric proteins, with altered length and sequences compared to the parental genomes. The potential selective advantage of such chimeric proteins is illustrated by recurring recombinations between ammonium permease *MEP2* alleles in a *S. cerevisiae* and *S. uvarum* hybrid during laboratory evolution under nitrogen-limited conditions (Dunn et al., 2013). The formation of chimeric ORFs has even led to the emergence of novel gene functions, as illustrated by the formation of a maltotriose transporter by recombination of three non-maltotriose transporter genes in *S. eubayanus* during laboratory evolution (Brouwers et al., 2018).

The predominant occurrence of recombinations within ORFs in the evolved isolates has also been observed in the genomes of brewing strains of *S. pastorianus*, which all share identical recombinations at the *ZUO1, MAT, HSP82*, and *XRN1*/*KEM1* loci (Hewitt et al., 2014; Walther et al., 2014; Okuno et al., 2016). These common recombinations suggest that all *S. pastorianus* isolates descend from a common ancestor (Monerawela and Bond, 2018). However, *S. pastorianus* strains may also have emerged from two independent hybridization events, as suggested by the presence of two genetically distinct groups within *S. pastorianus* (Dunn and Sherlock, 2008; Baker et al., 2015) Indeed, since identical recombinations have been observed in independent evolutions, identical recombinations might reflect parallel evolution due to a strong selective advantage under brewing-related conditions and/or a predisposition of specific loci for recombination (Dunham et al., 2002; Dunn et al., 2013). Of the recombination loci found in the present study, only *EFT1* and *MAT* loci were associated with recombinations in *S. pastorianus*. Moreover, the recombinations at these loci in the evolved isolates were different from those in *S. pastorianus* (Hewitt et al., 2014; Walther et al., 2014; Okuno et al., 2016; Monerawela and Bond, 2017). All interchromosomal recombinations observed in this study were unique. These results, obtained under brewing-related conditions, are consistent with the notion that recombination sites are largely aleatory and that all modern *S. pastorianus* strains share the same recombinations because they descend from a single hybrid ancestor.

Different recombination events resulted in the loss of heterozygosity, in four isolates each, of the right arm of *Se*CHRXV, including *SeSFL1*, and of the right arm of *Sc*CHRXI, including *ScMAL11*. These events directly affected two phenotypes relevant for brewing fermentation: calcium-dependent flocculation, which led to fast biomass sedimentation, and loss of maltotriose utilization. Biomass sedimentation can be strongly selected for in sequential batch bioreactors, as it increases the chance that cells are retained in the bioreactor during the emptying phase (Oud et al., 2013; Hope et al., 2017). A similar selective advantage is likely to have played a role in the early domestication of *S. pastorianus*, as sedimenting yeast remaining in fermentation vessels was more likely to be used in a next fermentation. Flocculation is a key characteristic of current lager-brewing yeasts (also referred to as bottom-fermenting yeasts), as it simplifies biomass separation at the end of the fermentation (Ferreira et al., 2010). The present study illustrates how this aspect of brewing yeast domestication can be rapidly reproduced under simulated laboratory conditions.

At first glance, loss of the ability to utilize maltotriose, an abundant fermentable sugar in wort, appears to be undesirable from an evolutionary perspective. However, as demonstrated in studies on laboratory evolution of *S. cerevisiae* in sequential batch cultures on sugar mixtures, the selective advantage of consuming a specific sugar from a sugar mixture correlates with the number of generations of growth on that sugar during each cultivation cycle (Wisselink et al., 2009; Verhoeven et al., 2018). *Saccharomyces* yeasts, including strain IMS0408 generally prefer glucose and maltose over maltotriose (Alves et al., 2008; Hebly et al., 2015; Brickwedde et al., 2017). As a consequence, maltotriose consumption from wort typically only occurs when growth has already ceased due to oxygen limitation and/or nitrogen source depletion, which results in few or no generations of growth on this trisaccharide. However, loss of maltotriose utilization in six isolates in two independent evolution experiments strongly suggests that loss of *ScMAL11* expression was not merely neutral but even conferred a selective advantage. These results are consistent with the existence of many *S. pastorianus* strains with poor maltotriose utilization and with the truncation of *ScMAL11* in all *S. pastorianus* strains, including good maltotriose utilizers (Vidgren et al., 2009; Gibson et al., 2013). In the latter strains, maltotriose utilization depends on alternative transporters such as Mty1 (Dietvorst et al., 2005; Salema-Oom et al., 2005). It is therefore unclear if a selective advantage of the loss of *ScMAL11* reflects specific properties of this gene or its encoded transporter or, alternatively, a general negative impact of maltotriose utilization under brewing-related conditions. In analogy with observations on maltose utilization by *S. cerevisiae*, unrestricted Mal11-mediated maltotriose-proton symport might cause maltotriose-accelerated death (Postma et al., 1990; Jansen et al., 2004). Alternatively, expression of the Mal11 transporter might compete with superior maltose transporters for intracellular trafficking, membrane integration and/or membrane space (Vidgren, 2010; Libkind et al., 2011). Indeed, laboratory evolution to obtain improved maltotriose utilization resulted in reduced maltose uptake in *S. pastorianus* (Brickwedde et al., 2017).

*Saccharomyces* hybrids are commonly applied for industrial applications such as lager beer brewing and wine fermentation (Naumov et al., 2001; González et al., 2006; Libkind et al., 2011). Recently, novel hybrids have been generated, that performed well under a broad range of industrially-relevant conditions (Bellon et al., 2011; Hebly et al., 2015; Lopandic et al., 2016; Krogerus et al., 2017; Nikulin et al., 2017; Peris et al., 2017). While the performance and phenotypic diversity of laboratory hybrids support their application in industrial processes, further strain development of such hybrids could improve their performance (Steensels et al., 2014; Krogerus et al., 2017). Especially in food and beverage fermentation processes, consumer acceptance issues largely preclude use of targeted genetic modification techniques. Laboratory evolution offers an interesting alternative strategy for “non-GMO” strain improvement (Bachmann et al., 2015). However, as exemplified by the loss, in independent laboratory evolution experiments, of *MAL11* and of the mitochondrial genome, mutations that yield increased fitness under simulated industrial fermentation conditions are not necessarily advantageous for industrial performance. Therefore, instead of faithfully reconstructing industrial conditions in the laboratory, laboratory evolution experiments should be designed to specifically select for desired phenotypes. For example, a recent study illustrated how maltotriose fermentation kinetics of an *S. pastorianus* hybrid could be improved by laboratory evolution in carbon-limited chemostats grown on a maltotriose-enriched sugar mixture (Brickwedde et al., 2017).

## Data Availability

The datasets generated by genome sequencing for this study can be found in the NCBI database (https://www.ncbi.nlm.nih.gov/) under the Bioproject PRJNA506072.

## Author Contributions

AG planned and executed the laboratory evolution, made single colony isolates, prepared genomic DNA and characterized ploidy and respiratory deficiency. AG, AvA, LK and LJ characterized flocculation and maltotriose utilization of the isolates. AG and MV performed the reverse engineering of deletions of *SFL1* and *MAL11* and characterized the reverse engineered phenotypes. AG and MvdB performed the bioinformatics analysis. AG, JP, and J-MD wrote the manuscript. AS, NB, and TA provided critical feedback throughout the study. J-MD and JP supervised the study. All authors read and approved the final manuscript.

### Conflict of Interest Statement

The authors declare that the research was conducted in the absence of any commercial or financial relationships that could be construed as a potential conflict of interest.

## Supplementary Material

Data Sheet S1 Mutations and mutation rates in the IMS0408-derived isolates obtained after laboratory evolution based on whole genome sequencing analysis. **(A)** SNPs identified in the isolates. **(B)** INDELs identified in the isolates. **(C)** chromosomal recombinations identified in the isolates. **(D)** whole chromosome aneuploidy identified in the isolates. **(E)** loss of mitochondrial DNA identified in the isolates. **(F)** mutation rates in the isolates.
