# Supplementary material for: Laboratory Evolution of a Saccharomyces cerevisiae × S. eubayanus Hybrid Under Simulated Lager-Brewing Conditions
Source: Front Genet. 2019 Mar 29;10:242. doi: 10.3389/fgene.2019.00242 (PMC6455053; doi:10.3389/fgene.2019.00242)
Supplement: Supplementary file 1 [file Presentation_1.pdf]

Supplementary Material

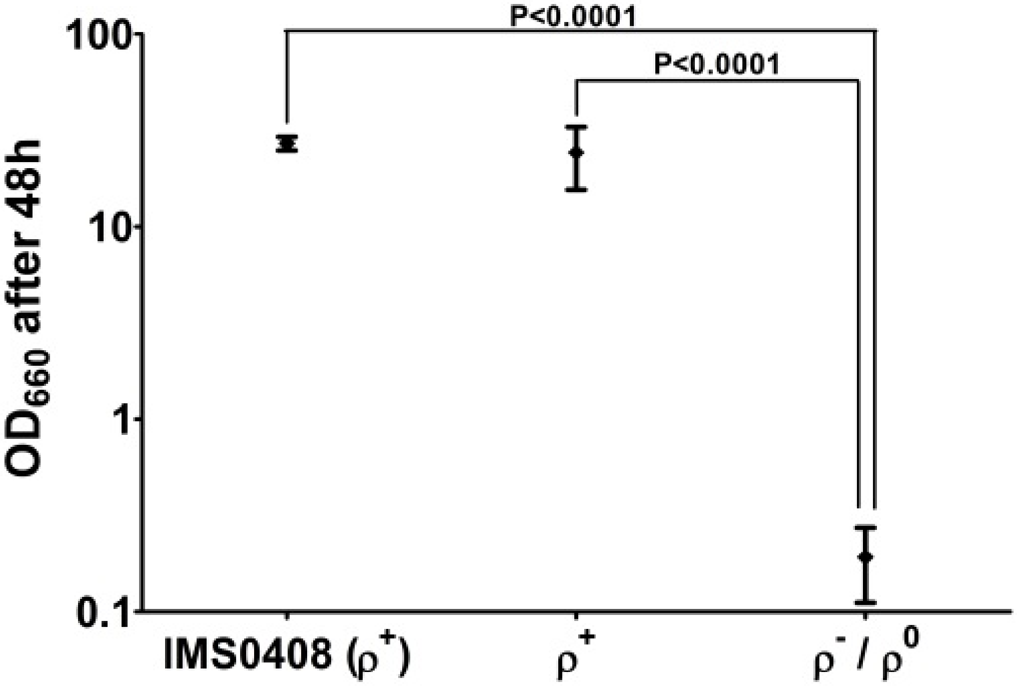

Supplementary Figure 1: Respiratory competence of IMS0408 and of isolates with (ρ^+) and without (ρ^- / ρ^0) a complete mitochondrial genome. Respiratory competence was assessed as optical density in a culture grown SM with 2% ethanol as sole carbon source. Cultures were inoculated at an OD_{660} of 0.2 and incubated for 72 h at 30 ˚C. Strains for which whole genome sequencing indicated absence of (part of) the mitochondrial DNA were grouped as ρ^-/ρ^0: IMS0541, IMS0546, IMS0549, IMS0550, IMS0551, IMS0552, IMS0566, IMS0567, IMS0601, IMS0606, IMS0608, IMS0610, IMS0611, IMS0616 and IMS0618. The final OD_{660} of cultures of ρ^-/ρ^0 strains was compared to those of the unevolved strain IMS0408 and of all evolved isolates with a complete mitochondrial genome (ρ^+). Error bars indicate standard deviation, data for strain IMS0408 are derived from triplicate cultures. Measured OD_{660} values for each strain are provided in Supplementary table S2. P-values were calculated using Student's t-test.

Supplementary Table 1: *Saccharomyces* strains used throughout this study.

Name	Species	Relevant Genotype	Source
IMS0408	*S. cerevisiae x eubayanus*	ρ^{+} *SeMATa/ScMATα scura3::KanMX/SeURA3 ScMAL11 ScSFL1 SeSFL1*	[1]
IMS0538	*S. cerevisiae x eubayanus*	ρ^{+} *SeMATa/ScMATα scura3::KanMX/SeURA3 ScMAL11 ScSFL1 SeSFL1*	LG12.1, 29 batches
IMS0539	*S. cerevisiae x eubayanus*	ρ^{+} *SeMATa/ScMATα scura3::KanMX/SeURA3 ScMAL11 ScSFL1 SeSFL1*	LG12.1, 29 batches
IMS0540	*S. cerevisiae x eubayanus*	ρ^{+} *SeMATa/ScMATα scura3::KanMX/SeURA3 ScMAL11 ScSFL1 SeSFL1 SeHDA2*^{A1651T} *SeMED2*$^{462+3N}$	LG12.1, 29 batches
IMS0541	*S. cerevisiae x eubayanus*	ρ^{0} *SeMATa/ScMATα scura3::KanMX/SeURA3 ScMAL11 ScSFL1 SeSFL1*	LG12.1, 29 batches
IMS0542	*S. cerevisiae x eubayanus*	ρ^{+} *SeMATa/ScMATα scura3::KanMX/SeURA3 ScMAL11 ScSFL1 SeSFL1 ScIQG1*^{A2069C} *SeKEX1*$^{1875-6N}$	LG12.1, 29 batches
IMS0543	*S. cerevisiae x eubayanus*	ρ^{+} Δ*Sc*(YKL032C-YKL054C) *SeMATa/ScMATα scura3::KanMX/SeURA3 ScMAL11 ScSFL1 SeSFL1*	LG12.1, 29 batches
IMS0544	*S. cerevisiae x eubayanus*	ρ^{+} Δ*Sc*(YKL032C-YKL054C) Δ*Sc*::*Se*(YLR154C-YLRend) *SeMATa/ScMATα scura3::KanMX/SeURA3 ScMAL11 ScSFL1 SeSFL1 SeSAC1*^{G1093C}	LG12.1, 29 batches
IMS0545	*S. cerevisiae x eubayanus*	ρ^{+} Δ*Sc*(YKL032C-YKL054C) *SeMATa/ScMATα scura3::KanMX/SeURA3 ScMAL11 ScSFL1 SeSFL1 SeNAF1*$^{1404-30N,1436-30N}$	LG12.1, 29 batches
IMS0546	*S. cerevisiae x eubayanus*	ρ^{-} Δ*Sc*(YKL032C-YKL054C) *SeMATa/ScMATα scura3::KanMX/SeURA3 ScMAL11 ScSFL1 SeSFL1*	LG12.1, 29 batches
IMS0547	*S. cerevisiae x eubayanus*	ρ^{+} Δ*Sc*(YKL032C-YKL054C) *SeMATa/ScMATα scura3::KanMX/SeURA3 ScMAL11 ScSFL1 SeSFL1*	LG12.1, 29 batches
IMS0548	*S. cerevisiae x eubayanus*	ρ^{+} Δ*Se*::*Sc*(YKLend-YKL057C) Δ*Sc*::*Se*(YKL057C-YKRend) *SeMATa/ScMATα scura3::KanMX/SeURA3 ScMAL11 ScSFL1 SeSFL1*	LG12.2, 29 batches
IMS0549	*S. cerevisiae x eubayanus*	ρ^{-} *SeMATa/ScMATα scura3::KanMX/SeURA3 ScMAL11 ScSFL1 SeSFL1*	LG12.2, 29 batches
IMS0550	*S. cerevisiae x eubayanus*	ρ^{-} *SeMATa/ScMATα scura3::KanMX/SeURA3 ScMAL11 ScSFL1 SeSFL1*	LG12.2, 29 batches
IMS0551	*S. cerevisiae x eubayanus*	ρ^{-} Δ*Sc*(YCLend-YCL067C) Δ*Sc*(YCR039C-YCRend) *SeMATa/ScMATα scura3::KanMX/SeURA3 ScMAL11 ScSFL1 SeSFL1*	LG12.2, 29 batches
IMS0552	*S. cerevisiae x eubayanus*	ρ^{-} Δ*Sc*::*Se*(YHLend-YHL023C) *SeMATa/ScMATα scura3::KanMX/SeURA3 ScMAL11 ScSFL1 SeSFL1*	LG12.2, 29 batches
IMS0553	*S. cerevisiae x eubayanus*	ρ^{+} *SeMATa/ScMATα scura3::KanMX/SeURA3 ScMAL11 ScSFL1 SeSFL1 SeNHX1*$^{1622+3N}$	LG30.1, 116 batches
IMS0554	*S. cerevisiae x eubayanus*	ρ^{+} Δ*Sc*::*Se*(YGR282C-YGRend) *SeMATa/ScMATα scura3::KanMX/SeURA3 ScSFL1 SeSFL1 ScCIS3**	LG30.1, 116 batches
IMS0555	*S. cerevisiae x eubayanus*	ρ^{+} *SeMATa/ScMATα scura3::KanMX/SeURA3 ScMAL11 ScSFL1 SeSFL1 SeBAT1*^{G1073A}, *ScMAL23*^{G422A}	LG30.1, 116 batches
IMS0556	*S. cerevisiae x eubayanus*	ρ^{+} 2x*Sc*(CHRVIII) *SeMATa/ScMATα scura3::KanMX/SeURA3 ScMAL11 SeSFL1 SeGMC1*^{G1579A}, *ScSFL1*^{T605A}	LG30.1, 116 batches
IMS0557	*S. cerevisiae x eubayanus*	ρ^{+} *SeMATa/ScMATα scura3::KanMX/SeURA3 ScSFL1 SeSFL1* Sc*MAL11*$^{G88T, A98G}$ *ScMDL2*^{C1451A} *ScMAL11*$^{93-1N}$ *Se*YNL247W$^{2062-1N}$	LG30.1, 116 batches
IMS0558	*S. cerevisiae x eubayanus*	ρ^{+} Δ*Sc*(YDR261C-YDR211W) Δ*Sc*::*Se*(YGR218C-YGRend) *SeMATa/ScMATα scura3::KanMX/SeURA3 ScSFL1*^{T605A} *SeSFL1*$^{96+1N}$	LG30.1, 116 batches
IMS0559	*S. cerevisiae x eubayanus*	ρ^{+} Δ*Se*::*Sc*(YOR133W-YORend) *SeMATa/ScMATα scura3::KanMX/SeURA3 ScMAL11 ScSFL1*^{T605A}	LG30.1, 116 batches
IMS0560	*S. cerevisiae x eubayanus*	ρ^{+} 2x*Se*(CHRX) Δ*Sc*(YDR261C-YDR211W) Δ*Se*::*Sc*(YOR063W-YORend) *SeMATa/ScMATα scura3::KanMX/SeURA3 ScMAL11 ScSFL1*^{T605A}	LG30.1, 116 batches
IMS0561	*S. cerevisiae x eubayanus*	ρ^{+} Δ*Se*::*Sc*(YBR275C-YBRend), Δ*Se*::*Sc*(YOR133W-YORend) *SeMATa/ScMATα scura3::KanMX/SeURA3 ScMAL11 ScSFL1*^{T605A}	LG30.1, 116 batches
IMS0562	*S. cerevisiae x eubayanus*	ρ^{+} Δ*Sc*::*Se*(YNL061C-YNL055C) Δ*Se*::*Sc*(YOR133W-YORend) *SeMATa/ScMATα scura3::KanMX/SeURA3 ScMAL11* ScSFL1T^{605A}	LG30.1, 116 batches
IMS0563	*S. cerevisiae x eubayanus*	ρ^{+} Δ*Sc*(YGR279C-YGRend)::*Se*(YMR305C-YMRend) *SeMATa/ScMATα scura3::KanMX/SeURA3 ScSFL1 SeSFL1 SeROG3*^{G191A} *SeMSS51*^{C448T} *ScRTP1*$^{874-64N}$	LG30.2, 117 batches
IMS0564	*S. cerevisiae x eubayanus*	ρ^{+} *SeMATa/ScMATα scura3::KanMX/SeURA3 ScMAL11 ScSFL1 SeSFL1 SeIZH3*^{A526G} *ScCST6*^{C807A}	LG30.2, 117 batches
IMS0565	*S. cerevisiae x eubayanus*	ρ^{+} Δ*Sc*::*Se*(CHRXIV) *SeMATa/ScMATα scura3::KanMX/SeURA3 ScSFL1 SeSFL1* Sc*MAL11*^{A1G}	LG30.2, 117 batches
IMS0566	*S. cerevisiae x eubayanus*	ρ^{-} *SeMATa/ScMATα scura3::KanMX/SeURA3 ScMAL11 ScSFL1 SeSFL1 ScERG6*^{C413T} *ScBUL1*^{G2110A} *Se*YBR238$^{C315-36N}$	LG30.2, 117 batches
IMS0567	*S. cerevisiae x eubayanus*	ρ^{-} Δ*Sc*::*Se*(YDR051C-YDRend) Δ*Sc*::*Se*(YGR271W-YGRend) *SeMATa/ScMATα scura3::KanMX/SeURA3 ScSFL1 SeSFL1 ScULP2*$^{1469+1N}$	LG30.2, 117 batches
IMS0594	*S. cerevisiae x eubayanus*	ρ^{+} *SeMATa/ScMATα scura3::KanMX/SeURA3 ScMAL11 ScSFL1 SeSFL1 SeELA1*$^{230+343N}$ *SeIRA2*$^{1402-1N}$ *ScFLO9**	LG12.1, 58 batches
IMS0595	*S. cerevisiae x eubayanus*	ρ^{+} 2x*Se*(CHRVIII) Δ*Se*::*Sc*(YOLend-YOL072W) *SeMATa/ScMATα scura3::KanMX/SeURA3 ScMAL11 ScSFL1 SeSFL1*	LG12.1, 58 batches
IMS0596	*S. cerevisiae x eubayanus*	ρ^{+} *SeMATa/ScMATα scura3::KanMX/SeURA3 ScMAL11 ScSFL1 SeSFL1 SeIRA2*^{C1376A} *ScYER188*^{WT28A}	LG12.1, 58 batches
IMS0597	*S. cerevisiae x eubayanus*	ρ^{+} Δ*Se*::*Sc*(YOLend-YOL057W) *SeMATa/ScMATα scura3::KanMX/SeURA3 ScMAL11 ScSFL1 SeSFL1 SeNUP1*^{C1205T} *ScPDC2*^{G372A}	LG12.1, 58 batches
IMS0598	*S. cerevisiae x eubayanus*	ρ^{+} *SeMATa/ScMATα scura3::KanMX/SeURA3 ScMAL11 ScSFL1 SeSFL1 SeSRT1*^{C359T} *SeASG1*$^{2488+3N}$	LG12.1, 58 batches
IMS0599	*S. cerevisiae x eubayanus*	ρ^{+} Δ*Sc*(YKL032C-YKL054C) Δ*Se*::*Sc*(YLR154C-YLRend) *SeMATa/ScMATα scura3::KanMX/SeURA3 ScMAL11 ScSFL1 SeSFL1*	LG12.1, 58 batches
IMS0600	*S. cerevisiae x eubayanus*	ρ^{+} Δ*Se*::*Sc*(YOLend-YOL075W) *SeMATa/ScMATα scura3::KanMX/SeURA3 ScMAL11 ScSFL1 SeSFL1 SeMSR1*^{A853C}	LG12.1, 58 batches
IMS0601	*S. cerevisiae x eubayanus*	ρ^{-} Δ*Sc*(YKL032C-YKL054C) *SeMATa/ScMATα scura3::KanMX/SeURA3 ScMAL11 ScSFL1 SeSFL1*	LG12.1, 58 batches
IMS0602	*S. cerevisiae x eubayanus*	ρ^{+} Δ*Sc*(YKL032C-YKL054C) *SeMATa/ScMATα scura3::KanMX/SeURA3 ScMAL11 ScSFL1 SeSFL1*	LG12.1, 58 batches

IMS0603	*S. cerevisiae x eubayanus*	ρ^+ Δ*Se*::*Sc*(YNLend-YNL123W) ΔSe::Sc(YOLend-YOL013C) ΔSc::Se(YOL013C-YOL006C) *SeMATa/ScMATα scura3::KanMX/SeURA3 ScMAL11 ScSFL1 SeSFL1*	LG12.1, 58 batches
IMS0604	*S. cerevisiae x eubayanus*	ρ^+ Δ*Sc*(YKL032C-YKL054C), Δ*Sc*::*Se*(YLR305C-YLRend) *SeMATa/ScMATα scura3::KanMX/SeURA3 ScMAL11 ScSFL1 SeSFL1 SeBET2*^{G550A}	LG12.2, 57 batches
IMS0605	*S. cerevisiae x eubayanus*	ρ^+ Δ*Sc*(YKL032C-YKL054C) *SeMATa/ScMATα scura3::KanMX/SeURA3 ScMAL11 ScSFL1 SeSFL1*	LG12.2, 57 batches
IMS0606	*S. cerevisiae x eubayanus*	ρ^- Δ*Se*(CHRVIII) Δ*Sc*(YKL032C-YKL054C) *SeMATa/ScMATα scura3::KanMX/SeURA3 ScMAL11 ScSFL1 SeSFL1 ScLRG1*^{C2277G}	LG12.2, 57 batches
IMS0607	*S. cerevisiae x eubayanus*	ρ^+ Δ*Sc*(YKL032C-YKL054C) *SeMATa/ScMATα scura3::KanMX/SeURA3 ScMAL11 ScSFL1 SeSFL1 SeFLO11**	LG12.2, 57 batches
IMS0608	*S. cerevisiae x eubayanus*	ρ^- Δ*Sc*(YKL032C-YKL054C) *SeMATa/ScMATα scura3::KanMX/SeURA3 ScMAL11 ScSFL1 SeSFL1*	LG12.2, 57 batches
IMS0609	*S. cerevisiae x eubayanus*	ρ^+ *SeMATa/ScMATα scura3::KanMX/SeURA3 ScMAL11 ScSFL1 SeSFL1*	HG12.1, 13 batches
IMS0610	*S. cerevisiae x eubayanus*	ρ^- *SeMATa/ScMATα scura3::KanMX/SeURA3 ScMAL11 ScSFL1 SeSFL1 Sc*YBR259W^{C833A}	HG12.1, 13 batches
IMS0611	*S. cerevisiae x eubayanus*	ρ^- *SeMATa/ScMATα scura3::KanMX/SeURA3 ScMAL11 ScSFL1 SeSFL1 ScFMP52*^{G406C}	HG12.1, 13 batches
IMS0612	*S. cerevisiae x eubayanus*	ρ^+ *SeMATa/ScMATα scura3::KanMX/SeURA3 ScMAL11 ScSFL1 SeSFL1*	HG12.1, 13 batches
IMS0613	*S. cerevisiae x eubayanus*	ρ^+ *SeMATa/ScMATα scura3::KanMX/SeURA3 ScMAL11 ScSFL1 SeSFL1 SeGIC2*^{C344G} *SeGCN2*$^{2274+18N,\ 2239+18N}$ *SeTRA1*$^{4421-25N}$	HG12.1, 13 batches
IMS0614	*S. cerevisiae x eubayanus*	ρ^+ Δ*Se*(YAR050W-YARend)::*Se*(YALend-YAL063C) *SeMATa/ScMATα scura3::KanMX/SeURA3 ScMAL11 ScSFL1 SeSFL1*^{C1390T}	HG12.2, 16 batches
IMS0615	*S. cerevisiae x eubayanus*	ρ^+ *SeMATa/ScMATα scura3::KanMX/SeURA3 ScMAL11 ScSFL1 SeSFL1 ScCAC2*^{A994T}	HG12.2, 16 batches
IMS0616	*S. cerevisiae x eubayanus*	ρ^- *SeMATa/ScMATα scura3::KanMX/SeURA3 ScMAL11 ScSFL1 SeSFL1 ScATG1*^{A434C}	HG12.2, 16 batches
IMS0617	*S. cerevisiae x eubayanus*	ρ^+ *SeMATa/ScMATα scura3::KanMX/SeURA3 ScMAL11 ScSFL1 SeSFL1*^{C1390T}	HG12.2, 16 batches
IMS0618	*S. cerevisiae x eubayanus*	ρ^- *SeMATa/ScMATα scura3::KanMX/SeURA3 ScMAL11 ScSFL1 SeSFL1 ScALR1*^{G1645T}	HG12.2, 16 batches
IMX1824	*S. cerevisiae x eubayanus*	ρ^+ *SeMATa/ScMATα scura3::KanMX/SeURA3 ScMAL11 SeSFL1 mTurquoise2::ΔScSFL1*	Cas9 deletion in IMS0408
IMX1825	*S. cerevisiae x eubayanus*	ρ^+ *SeMATa/ScMATα scura3::KanMX/SeURA3 ScMAL11 ScSFL1 Venus::ΔSeSFL1*	Cas9 deletion in IMS0408
IMX1826	*S. cerevisiae x eubayanus*	ρ^+ *SeMATa/ScMATα scura3::KanMX/SeURA3 ScMAL11 mTurquoise2::ΔScSFL1, Venus::ΔSeSFL1*	Cas9 deletion in IMS0408
IMX1698	*S. cerevisiae x eubayanus*	ρ^+ *SeMATa/ScMATα scura3::KanMX/SeURA3 ScMAL11 ScSFL1 SeSFL1* mVenus::Δ*ScMAL11*	Cas9 deletion in IMS0408

1 Hebly, M. *et al.* S. cerevisiae× S. eubayanus interspecific hybrid, the best of both worlds and beyond. *FEMS yeast research* **15** (2015).

Supplementary table 2: Oligonucleotides used in this study. Primers 12989, 12990 and 13564-13567 were ordered as *de novo* synthesized HPLC-purified oligonucleotides at Sigma-Aldrich and gRNA$_{ScSFL1}$ and gRNA$_{SeSFL1}$ were ordered *de novo* synthesized plasmids at GeneArt (ThermoFisher Scientific).

name	Sequence (3' to 5')	Purpose
12989	TTGGTGTTTCTTTCTGATGCTACATAGAAGAACATCAAACAACTAAAAAAATAGTATAATACA CACTGGCTTAAGATGAC	Insertion Venus in *MAL11*
12990	TTTGGGAGCAGTCAAAGGGATTCCTTATTTCTTCCAAAAAAAAAAAAAACAACCCTTTTACCGTC TCATGCTCAGC	Insertion Venus in *MAL11*
13564	ACTTTTTCAGCTAGCAAGAAGGATCTCTTTTTAAACACTCTATACAGGTGCACACAAAGGACCA GATGTCAACACAGCTAC	Insertion mTurquoise2 in *ScSFL1*
13565	CGGAGTTGGTAAAAATATAGTTATAATCACAAGGATCAGGAGGAAAAGAAAAAAAAAGTGATT TATCGTCTCACATCCAGC	Insertion mTurquoise2 in *ScSFL1*
13566	GTAAGAAAGCAGAAAAAAAAAGAGAAAAAAAGAAAACGATCTCGACTAGAGTGACGGGTTGAC ACACTGGCTTAAGATGAC	Insertion Venus in *SeSFL1*
13567	AAAATGGAAGGGTGAAAATGGAAGGAAGAAGTGGAAAGTGAAAAATGAAAAATACAATGTC GTCTCATGCTCAGC	Insertion Venus in *SeSFL1*
gRNA *ScSFL1*	GGTCTCGCAAAATTCATCTGATGAGTCCGTGAGGACGAAACGAGTAAGCTCGTCATGAATATC GCATTTTGTGGGTTTTAGAGCTAGAAATAGCAAGTTAAAATAAGGCTAGTCCGTTATCAACTTG AAAAAGTGGCACCGAGTCGGTGCTTTTGGCCGGCATGGTCCCAGCCTCCTCGCTGGCGCCGGC TGGGCAACATGCTTCGGCATGGCGAATGGGACACAGCGAGACC	Sequence ordered in plasmid pUD711
gRNA *SeSFL1*	GGTCTCGCAAATAACAACTGATGAGTCCGTGAGGACGAAACGAGTAAGCTCGTCTTGTTATAG TCACGGATCGAGTTTTAGAGCTAGAAATAGCAAGTTAAAATAAGGCTAGTCCGTTATCAACTT GAAAAAGTGGCACCGAGTCGGTGCTTTTGGCCGGCATGGTCCCAGCCTCCTCGCTGGCGCCGG CTGGGCAACATGCTTCGGCATGGCGAATGGGACACAGCGAGACC	Sequence ordered as plasmid pUD712

Supplementary Table 3: Genome size of IMS0408 and the evolved isolates as measured by flow cytometry. The average and standard deviation of 3 biological replicates is shown.

	Genome Size	
Strain	Average	Standard deviation
IMS0408	21,3	1,9
IMS0538	21,6	2,1
IMS0539	21,7	2,2
IMS0540	23,5	2,1
IMS0541	20,3	2,4
IMS0542	21	2
IMS0543	22,7	2
IMS0544	22,1	1,9
IMS0545	21,7	1,8
IMS0546	22,1	1,7
IMS0547	22,7	1,5
IMS0548	20,9	1,7
IMS0549	20	2
IMS0550	18,6	2
IMS0551	19,8	2,1
IMS0552	18,5	1,9
IMS0553	21,6	1,4
IMS0554	21,2	1,2
IMS0555	20,9	1,5
IMS0556	21,7	1,6
IMS0557	21,2	1,4
IMS0558	21,2	1,4
IMS0559	21,2	1,6
IMS0560	22,4	1,2
IMS0561	20,9	1,3
IMS0562	21,3	1,3
IMS0563	21,6	1,3
IMS0564	20,6	1,3
IMS0565	20,1	1,3
IMS0566	17,6	1,7
IMS0567	20,6	1,4
IMS0594	20,4	1,4
IMS0595	22,3	1,2
IMS0596	20,4	1,5
IMS0597	20,9	1,4
IMS0598	20,5	1,2
IMS0599	22,2	1,2
IMS0600	21	1,2
IMS0601	21,8	1,1
IMS0602	21,4	1,3
IMS0603	21,9	1,1
IMS0604	20,3	1,3
IMS0605	20,9	1,2
IMS0606	18,6	1,6
IMS0607	20,5	1,3
IMS0608	19,4	1,3
IMS0609	19,8	1,1
IMS0610	19,2	1,4
IMS0611	19	1,3
IMS0612	19,5	1,1
IMS0613	19,6	1,1
IMS0614	19,5	1,3
IMS0615	19,6	1,3
IMS0616	19,2	1,3
IMS0617	19,5	1,3
IMS0618	18,5	1,4
